# Surface Roughness Enhances Self-Nucleation of High-Density Polyethylene Droplets Dispersed within Immiscible Blends

**DOI:** 10.1021/acs.macromol.1c02487

**Published:** 2022-02-11

**Authors:** Seif Eddine Fenni, Maria Rosaria Caputo, Alejandro J. Müller, Dario Cavallo

**Affiliations:** † Dipartimento di Chimica e Chimica Industriale, Università degli studi di Genova, via Dodecaneso 31, 16146 Genova, Italy; ‡ Polymat and Department of Polymers and Advanced Materials: Physics, Chemistry and Technology, Faculty of Chemistry, University of the Basque Country UPV/EHU, Paseo Manuel de Lardizabal 3, 20018 Donostia-San Sebastián, Spain; § IKERBASQUE, Basque Foundation for Science, Plaza Euskadi 5, 48009 Bilbao, Spain

## Abstract

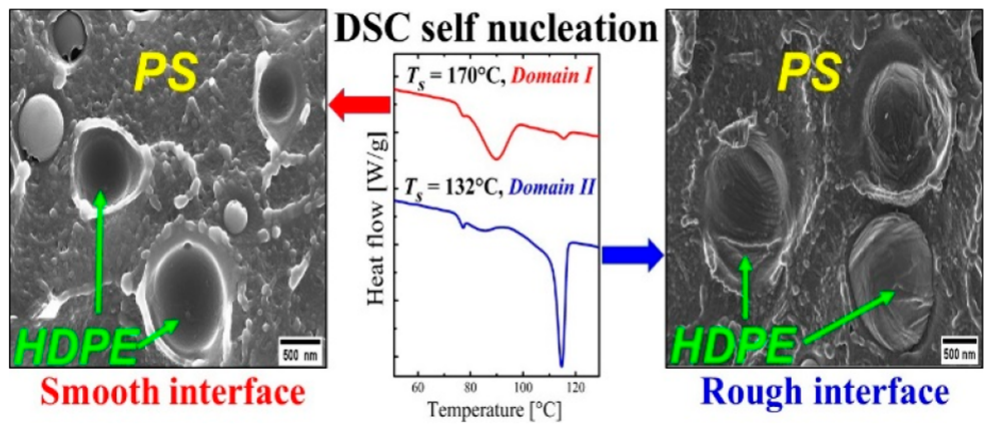

Highly linear or high-density polyethylenes (HDPEs) have an intrinsically high nucleation density compared to other polyolefins. Enhancing their nucleation density by self-nucleation is therefore difficult, leading to a narrow self-nucleation *Domain* (i.e., the so-called *DomainII* or the temperature *Domain* where self-nuclei can be injected into the material without the occurrence of annealing). In this work, we report that when HDPE is blended (up to 50%) with immiscible matrices, such as atactic polystyrene (PS) or Nylon 6, its self-nucleation capacity can be greatly increased. In addition, temperatures higher than the equilibrium melting temperature of the HDPE phase are needed to erase the significantly enhanced crystalline memory in the blends. Morphological evidence gathered by Scanning and Transmission Electron Microscopies (SEM and TEM) indicates that these unexpected results can be explained by the modification of the interface between blend components. The filling of the solid HDPE surface asperities by the low viscosity polystyrene during heating to the self-nucleation temperature, or the crystallization of the matrix in the case of Nylon 6, enhances the interface roughness between the two polymers in the blends. Such rougher interfaces can remarkably increase the self-nucleation capacity of the HDPE phase via surface nucleation.

## 1 Introduction

Polymer blending is a useful way to prepare polymer systems that exhibit an attractive combination of the properties of the neat polymer components.^1,2^ As an outcome of the blending process, two categories of polymer blends can be obtained, i.e., miscible and/or immiscible blends. In immiscible blends, mixing a small amount of a semicrystalline polymer with a second immiscible polymer (either amorphous or semicrystalline) often leads to the formation of a sea-island morphology, in which microdomains (MDs) or droplets of the minor crystalline phase will be dispersed in the matrix of the major phase.^3,4^ The crystallization behavior and superstructure of the mixed polymers, in the case of semicrystalline component(s), are affected by the blending process.^3−5^ The observed change is mainly related to the nucleation behavior (including both the mechanism and kinetics) of the minor phase. Given the fact that droplets are typically small (i.e., few micrometers or less), nucleation can be considered as the rate-determining step in the overall crystallization process.^6−8^

Fractionated crystallization is often encountered during crystallization of the minor semicrystalline component in immiscible blends. The fractionated crystallization phenomenon has been the subject of a recent comprehensive review, see ref (6). It arises because of the lack of active heterogeneities in every single droplet.^6^ Hence, during melt-crystallization, a different set of droplets will crystallize at different degrees of supercooling. Droplets that contain at least one highly active heterogeneity will crystallize at a crystallization temperature similar or close to that of the neat component, while the other sets of droplets will crystallize at larger supercoolings. Clean droplets or droplets with inert heterogeneities will crystallize at the highest supercooling via surface nucleation or homogeneous nucleation.^6,9,10^

Surface nucleation was found to play an important role in the crystallization of immiscible blends (both for the matrix and the dispersed micro-domains).^5,9^ Several researchers reported that surface nucleation could initiate from solid polymer surfaces as well as from molten surfaces/polymers.^11,12^ Fenni et al.^12^ reported the nucleation effect of molten poly(ε-caprolactone) (PCL) and molten poly(butylene succinate) (PBS) on poly(lactic acid) (PLA) self-assembled droplets in their 45/10/45 PCL/PLA/PBS immiscible ternary blend. While in another work, Fenni et al.^13^ showed that in 45/10/45 PLA/PCL/PBS ternary blends, the PBS continuous phase was able to nucleate at the surface of the previously crystalline PLA. Several factors were claimed to control and affect surface nucleation, such as polarity of the polymers,^14^ affinities between different components,^12^ states of the interface, and surface roughness.^7,15^ Regarding this last factor, Dalnoki-Veress and Carvalho found a direct correlation between the nucleation mechanism of the droplets and the roughness of the substrate in their polystyrene/poly(ethylene oxide) (PS/PEO) systems.^15^ Carmeli et al.^7^ reported a clear change in the crystallization kinetics of the dispersed high-density polyethylene (HDPE) droplets induced by changing the surface roughness of the polypropylene (PP) matrix via self-nucleation (SN).

Self-nucleation (SN), which is considered as one of the possible nucleation mechanisms in polymers, is the process of the production of self-nuclei and/or self-seeds by applying a specific thermal protocol based on partial melting of the polymer, i.e., either by using lower melting temperature or shorter melting times. SN is a useful strategy to promote polymer nucleation. However, the exact nature of the produced self-nuclei is not univocally assessed.^16−18^ Müller et al.^17,18^ have extensively investigated the self-nucleation of polymers, and recently, its application, the major experimental variables that could affect it, its interpretations, and the recent experimental combined techniques used to characterize and interpret it have been summarized.^18^

Three self-nucleation *domains* can be defined based on the DSC cooling and heating scans during a SN protocol.^16^*Domain I* (*DI*) or the isotropic melt *domain* is encountered when the crystallization behavior of the polymer is driven exclusively by high-temperature-resistant heterogeneous nuclei. *Domain II* (*DII*) or the self-nucleation *domain* is the temperature region in which the applied self-nucleation temperature (*T*_s_) is (i) low enough to leave some self-nuclei, which will accelerate the crystallization during the subsequent cooling scan, but (ii) not enough to leave any crystal fragment that anneals and affects the final melting behavior of the polymer. The lowest temperature in *Domain II* is defined as the ideal self-nucleation temperature (*T*_s ideal_). It is the temperature at which a maximum increase of the crystallization temperature, during subsequent cooling, is recorded while no change in the melting behavior is observed. Any further decrease of the *T*_s_ below the *T*_s ideal_ will lead to annealing and thickening of some crystal fragments, leading to the appearance of annealing melting peaks at higher temperature with respect to the conventional melting point of the polymer. This self-nucleation temperature range is called *Domain III* (*DIII*). Müller et al.^17−19^ proposed a further division of the *DII* into two sub-domains, i.e., (a) the *melt-memory subdomain* (*DIIa*) that occurs at the higher temperature range of the *DII*, where the applied *T*_s_ is high enough to melt crystals without fully erasing the melt memory, and (b) the *self-seeding subdomain* (*DIIb*) in which the applied temperatures are capable to melt the polymer crystals but low enough to leave crystal fragments called self-seeds.

Unlike most of the polymers that exhibit three self-nucleation *domains*, high-density polyethylene (HDPE) exhibits a very peculiar self-nucleation behavior. In fact, no clear accordance about the number of SN *domains* in HDPE was reached up to date. Indeed, in several works, the HDPE homopolymer and the polyethylene block within copolymers presented only *DI* and *DIII*.^20,21^ Trujillo et al.^20^ reported a direct transition between *DI* and *DIII* in their HDPE homopolymer. The authors attributed the total absence of the *Domain II* to the extremely high number of active heterogeneities that originally exist in the HDPE, which hinder SN from showing any further increase in the nucleation density. On the other hand, additional works reported a very narrow *DII* for the HDPE and polyethylene in copolymers.^7^

Interestingly, Alamo et al.^22^ reported a strong self-nucleation effect, even at temperatures above the equilibrium melting point (*T*_m_°) in random ethylene copolymers. Such effect is not observed in linear homopolymers and is thus attributed to the complex melt topology created by sequence length selection during copolymer crystallization.^22^

Self-nucleation was widely used to investigate the crystallization of droplets in immiscible blends. Among other applications, SN was applied in order to overcome the fractionated crystallization of the minor crystalline phase in immiscible blends.^23,24^ In the present work, SN of the HDPE dispersed droplets in immiscible blends, either in amorphous or semicrystalline matrices, has been investigated. It is shown for the first time that the self-nucleation *Domain DII* of HDPE can be largely extended only by dividing the HDPE into micro-domains in contact with foreign matrices interfaces.

## 2 Materials and Experiments

### 2.1 Materials

Two high-density polyethylene (HDPE) grades were used in order to demonstrate the generality of the findings. The first HDPE (MB7541) was a commercial grade provided by Borealis, and it was characterized by a melting point (*T*_m_) of around 130 °C, a melt flow rate (MFR) of about 4 g/10 min, and a density of 0.954 g/cm^3^. It is indicated in the following as HDPE-1. A second HDPE (Rigidex HD6070EA), a commercial grade provided by Ineos Polyolefins, has a melting point (*T*_m_) of around 133 °C, a melt flow rate (MFR) of 7.6 g/10 min, and a density of 0.960 g/cm^3^. The second HDPE is coded as HDPE-2.

Polystyrene (PS), with an MFR of 1.3 g/10 min, was purchased from Sigma Aldrich. It had a density of 1.04 g/cm^3^, a weight-average molecular weight (*M*_w_) of 350 kg/mol, and a dispersity (*M*_w_/*M*_n_) of 2.05.

The Nylon 6 used in this study was Durethan B30S provided by LANXESS. It had a density of 1.14 g/cm^3^ and a melting point of around 220 °C.

We note that HDPE-1 was blended with PS, while HDPE-2 was used for the blend with Nylon 6.

### 2.2 Blend Preparation

PS/HDPE-1 blends were prepared in a Brabender-type internal mixer. Melt mixing was performed at 200 °C using a rotor speed of 100 rpm for 10 min. Meanwhile, the Nylon 6/HDPE-2 blend was prepared in a Collin ZK25 co-rotating twin screw extruder-kneader, with a rotor speed of 180 rpm and a mixing temperature of 230 °C. All the prepared blends are summarized in Table 1.

**Table 1:** Composition of the Prepared Samples

sample	HDPE (wt %)	PS (wt %)	Nylon 6 (wt %)
HDPE-1	100		
Nylon 6			100
90/10 PS/HDPE-1	10	90	
85/15 PS/HDPE-1	15	85	
80/20 PS/HDPE-1	20	80	
70/30 PS/HDPE-1	30	70	
50/50 PS/HDPE-1	50	50	
90/10 Nylon 6/HDPE-2	10		90

### 2.3 Blend Characterization

#### 2.3.1 SEM Analysis

The morphology of the fractured surface of the different blends was investigated using a field-emission scanning electron microscope (Supra 40 VP model, Zeiss, Germany) at an accelerating voltage of 5 kV.

Two methods were applied during the preparation of the investigated samples. In the first one, specimens were directly submerged in liquid nitrogen for 30 min and fractured cryogenically. Meanwhile, in the second method, samples were subjected to the thermal protocol shown in Figure S1 prior to the cryogenic fracture. All samples were finally thinly sputter-coated with carbon using a Polaron E5100 sputter-coater.

The number-average (*d*_n_) and volume-average (*d*_v_) diameters were calculated using the equations shown in ref (23) by measuring around 200 droplets from different regions of the samples.

#### 2.3.2 TEM Analysis

To better clarify the morphological structure of some of the samples involved in this study, a TEM analysis was performed. Since the samples are essentially composed of carbon and hydrogen, they do not have much difference in terms of electron density; therefore, to be observed by TEM, they must undergo the staining process. For this purpose, a RuO_4_ solution was used. Thin strips of samples were put into this solution for 16 h. Afterward, ultrathin sections were cut at −90 °C with a diamond knife on a Leica EMFC6 cryo-ultramicrotome device. The ultrathin sections of 90 nm thickness were mounted on 200 mesh copper grids. Finally, they were examined using a TECNAI G2 20 TWIN TEM equipped with a LaB_6_ filament operating at an accelerating voltage of 120 kV.

#### 2.3.3 Thermal Behavior by Means of DSC

##### 2.3.3.1 Nonisothermal Analyses

Different thermal characterizations were done in two different laboratories and using two different DSCs. Neat HDPE-1 and PS/HDPE-1 blends were characterized using a DSC1 STARe System (Mettler Toledo, Switzerland). All measurements were performed using sample masses in the range of 3–5 mg and under a continuous nitrogen flow (20 mL/min). In this DSC analysis, neat HDPE-1 and PS/HDPE-1 blends were first heated from room temperature to 170 °C at 10 °C/min and held at 170 °C for 3 min, to erase the thermal history of the HDPE-1 component. The samples were then cooled at a cooling rate of 10 °C/min from 170 to 20 °C while the cooling scan was recorded. Finally, a second heating scan at a heating rate of 10 °C/min was performed and acquired.

On the other hand, a PerkinElmer Pyris I DSC equipped with an Intracooler 2P was employed to characterize the thermal properties of neat Nylon 6 and the Nylon 6/HDPE-2 blend.

All the experiments were performed under an ultrapure nitrogen flow, and the instrument was calibrated with indium and tin standards. Samples of 10 mg for the blend, i.e., 1 mg for the neat PE and neat Nylon 6 (with respect to the composition in the total blend), were used. Measurements were performed by placing the samples in sealed aluminum pans. Before being subjected to heat treatments, the samples were kept in a vacuum oven at 100 ° C overnight, to eliminate any trace of moisture absorbed during storage. Nonisothermal experiments of neat polymers and the blends were carried out following the same thermal protocol but with different temperatures. The neat HDPE-2 was first heated at 20 °C/min up to 180 °C and left at 180 °C for 3 min to erase the thermal history; then, it was cooled at 20 °C/min down to 25 °C and held for 1 min at this temperature. Finally, it was reheated at 20 °C/min up to 180 °C. The same method was used for neat Nylon 6 and Nylon 6/HDPE-2 but employing a maximum melt temperature of 250 °C since Nylon 6 has a higher melting temperature than HDPE-2.

##### 2.3.3.2 Self-Nucleation Experiments (SN)

PS/HDPE-1 samples were analyzed using the self-nucleation procedure described below.^16−18^

(1) The crystalline history was erased by melting the sample at 170 °C for 3 min (40 °C above the melting point of the neat HDPE-1 component).
(2) The sample was cooled to 0 °C, at a cooling rate of 10 °C/min, to create a standard crystalline state.
(3) Partial (or complete) melting of the sample was performed by heating at 10 °C/min to the SN temperatures (*T*_s_) and holding it there for 5 min.
(4) Cooling to 0 °C was performed, at a cooling rate of 10 °C/min, to crystallize the sample and detect the effect of the annealing at the different SN temperatures.
(5) A final heating scan from 0 to 170 °C of the recrystallized sample was performed at a rate of 10 °C/min. Regarding the Nylon 6/HDPE-2 blend, a relatively different thermal protocol was applied in which the melting point used in steps (1) and (5) was set at 200 °C while the applied scan (both cooling and heating) rate was 20 °C/min instead of the 10 °C/min used for the PS/HDPE-1 blends.

## 3 Results and Discussion

### 3.1 Morphological Characterization

**Figure 1:**
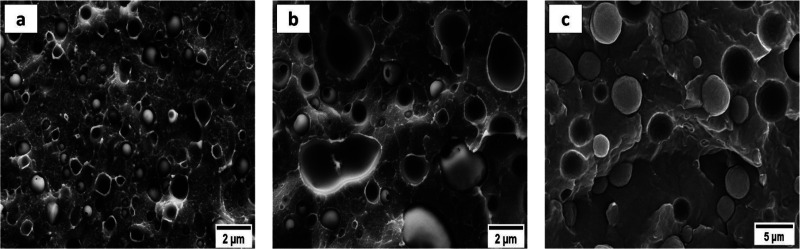
Morphologies of different binary blends. (a) 90/10 PS/HDPE-1, (b) 80/20 PS/HDPE-1, and (c) 90/10 Nylon 6/HDPE-2.

Figure 1 and Figure S1 (in the SI) present micrographs of the cryogenically fractured surface of all the investigated blends. In addition to the 50/50 PS/HDPE-1 blend, which exhibits a co-continuous morphology, and the 70/30 PS/HDPE-1, which displays a mixture of sea-island and co-continuous morphologies (see Figure S2), all the other blends exhibit a sea-island morphology in which the minor HDPE phase is present in the form of dispersed droplets or micro-domains (MDs) in the amorphous PS or semicrystalline Nylon 6 matrices. The morphology of each blend confirms the immiscibility of the studied systems.

Table 2 reports the size of dispersed micro-domains in the various blends. The size of the dispersed droplets changes with the HDPE-1 content and with the type of matrix. For instance, by increasing the HDPE-1 content from 10 to 20 wt % in the PS/HDPE-1 blends, the size (*d*_n_/*d*_v_) of the HDPE-1 droplets increases from 0.63/0.92 to 1.66/2.61 μm, respectively. On the other hand, a larger micro-domain size was found in the 90/10 Nylon 6 /HDPE-2 blend, even though the HDPE-2 content was only 10 wt %.

**Table 2:** Number-Average (*d*_n_) and Volume-Average Diameters (*d*_v_) and Dispersity (*D*) of the Investigated Blends

blend	*d*_n_ [μm]	*d*_v_ [μm]	*D*
90/10 PS/HDPE-1	0.63	0.92	1.46
80/10 PS/HDPE-1	1.66	2.61	1.57
90/10 Nylon 6/HDPE-2	3.23	3.61	1.12

The obtained difference in the size of the HDPE micro-domains at equivalent weight contents, in the PS/HDPE-1 and Nylon 6/HDPE-2 blends, should be attributed to the differences in the melt–viscosity ratio, shear rate, interfacial tension between the two components, or processing conditions.^25^

As previously known, the size of the dispersed micro-domains in immiscible blends is crucial and has a strong effect on the final crystallization behavior of the minor phase.^13,23,24,26−28^

### 3.2 DSC Nonisothermal Analysis

**Figure 2:**
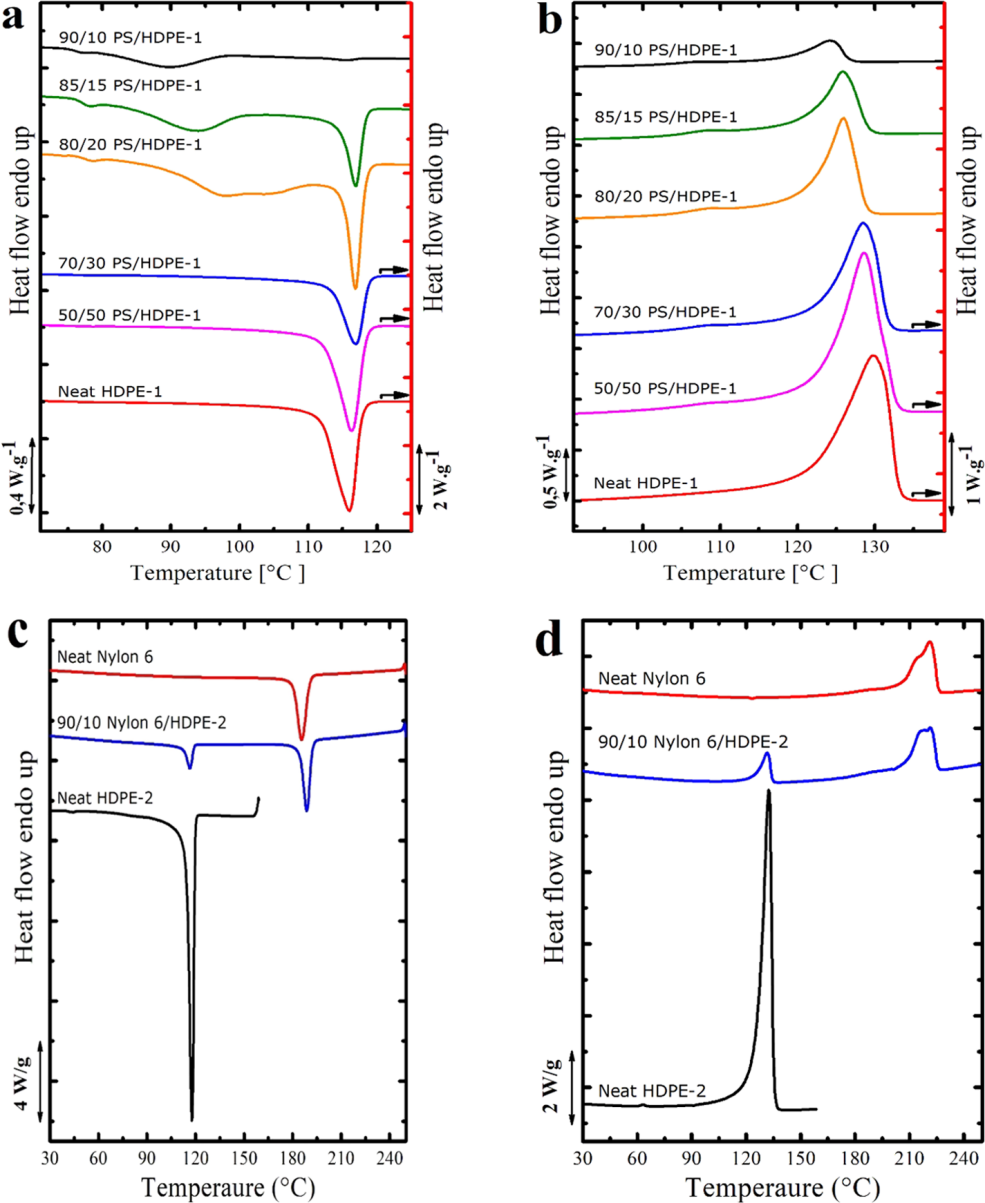
(a) DSC cooling scans and (b) subsequent DSC heating scans of the neat HDPE-1 and PS/HDPE-1 blends at a cooling and heating rate of 10 °C/min. (c) DSC cooling scans and (d) subsequent DSC heating scans of the neat HDPE-2 and Nylon 6/HDPE-2 blends at a scan rate of 20 °C/min.

Results of the DSC standard cooling and heating scans are shown in Figure 2 and Figure S3. It should be noted that PS/HDPE-1 blends have been analyzed using a scan rate of 10 °C/min (Figure 2a,b and Figure S3), while for the system Nylon 6/HDPE-2, a scan rate of 20 °C/min has been applied (Figure 2c,d). The crystallization temperatures (*T*_c_) and melting temperatures (*T*_m_) of the HDPE phase in all investigated blends are summarized in Table S1 of the Supporting Information (SI). At first, neat components will be considered. Neat HDPE-1 displays one single sharp crystallization peak at around 116 °C and melts at around 130 °C. Neat Nylon 6 exhibits a crystallization peak at about 186 °C and melts with a broad peak at around 221 °C. The neat PS, which is amorphous, has a *T*_g_ at around 105 °C.

Regarding the HDPE phase in the various blends, a clear correlation between the crystallization behavior and the morphology is found. First, for PS/HDPE-1 blends where the sea-island morphology is still preserved (blends with an HDPE-1 content of less than 30 wt %), fractionated crystallization is observed. For instance, for the 90/10 PS/HDPE-1 blend, multiple crystallization events at different supercoolings (at 115.7, 90, and 77.5 °C) are encountered. As discussed in the Introduction, during cooling from the melt, each set of droplets will crystallize at different supercooling, e.g., the droplets that contain highly active impurities will crystallize at a low supercooling (i.e., at the temperature at which the bulk polymer crystallizes). In comparison, micro-domains containing less active heterogeneities solidify at higher supercooling. Clean droplets will crystallize at a very high supercooling via surface nucleation or homogeneous nucleation.^3,9,10,29−32^ Another reason that could lead to this crystallization behavior is the migration of more active impurities/heterogeneities from the HDPE-1 phase to the PS during melt mixing. This will result in a meaningful lowering in the nucleation rate inside the dispersed HDPE-1 droplets.^31,33,34^ In the present case, for the 90/10 PS/HDPE-1 blend, only a limited number of droplets crystallize at a high crystallization temperature (namely, at 115.6 °C), and their crystallization enthalpy is very low (nearly negligible with respect to the total crystallization enthalpy of the HDPE-1 phase). On the other hand, the largest portion of the HDPE-1 micro-domains solidifies at a higher supercooling: a main large crystallization peak is observed at 90 °C, and its crystallization enthalpy is the largest among all peaks. Lastly, a third crystallization event occurred at the highest supercooling (that is, at 77 °C). This should be attributed to droplets free from impurities that crystallize, most probably, via surface nucleation mechanism (homogeneous nucleation is excluded because the crystallization temperature of this set of micro-domains is well above the *T*_g_ of HDPE-1).^35^

By increasing the HDPE-1 content in the blends (in the 85/15 and 80/20 PS/HDPE-1 blends), the enthalpy of the high-temperature crystallization peak increases at the expense of the low-temperature crystallization peaks. The crystallization temperature of the low-temperature crystallization peaks, as well, is found to shift toward higher temperatures. The observed changes are attributed to the increase in the micro-domain sizes, which leads to a higher number of droplets containing active heterogeneities. Similar results, in which fractionated crystallization of the HDPE phase was observed, have been previously reported for the systems PS/HDPE,^24,36,37^ PET/HDPE,^38^ and PMMA/HDPE.^39^

In blends where the HDPE-1 content is equal to or above 30 wt %, the co-continuous morphology (or a mixture of co-continuous and sea-island morphologies) is observed and the size of the HDPE-1 phases is large enough; hence, a bulk-like crystallization behavior is observed.

Concerning the 90/10 Nylon 6/HDPE-2 system and even though the HDPE-2 is presented only in 10 wt %, a bulk-like crystallization behavior is observed. The obtained bulk-like crystallization could be attributed to the larger size of the HDPE-2 micro-domains, which may allow most of the HDPE-2 droplets to contain at least one highly active impurity. Another factor that could be the reason behind this bulk-like crystallization behavior is the presence of the previously crystallized Nylon 6 interface, which can lower the energy barrier needed for nucleation and hence accelerate the nucleation step and the overall crystallization rate of the dispersed droplets.

The DSC melting traces of the HDPE-1 phase in the PS/HDPE-1 blends are shown in Figure 2b. It is clear that the melting point of the HDPE-1 phase decreases with the HDPE-1 content in the blend. The obtained results are logical because the lower the HDPE-1 content, the lower the size of the micro-domains, which in turn will be reflected in the size and thickness of the formed lamellae. Hence, a lower melting point will be recorded. In the case of the Nylon 6/HDPE-2 blend (see Figure 2d), the HDPE-2 phase presents a melting point very similar to the one of neat HDPE-2; thus, the corresponding melting temperature has not changed.

### 3.3 Self-Nucleation

#### 3.3.1 Self-Nucleation of the Neat HDPE-1

**Figure 3:**
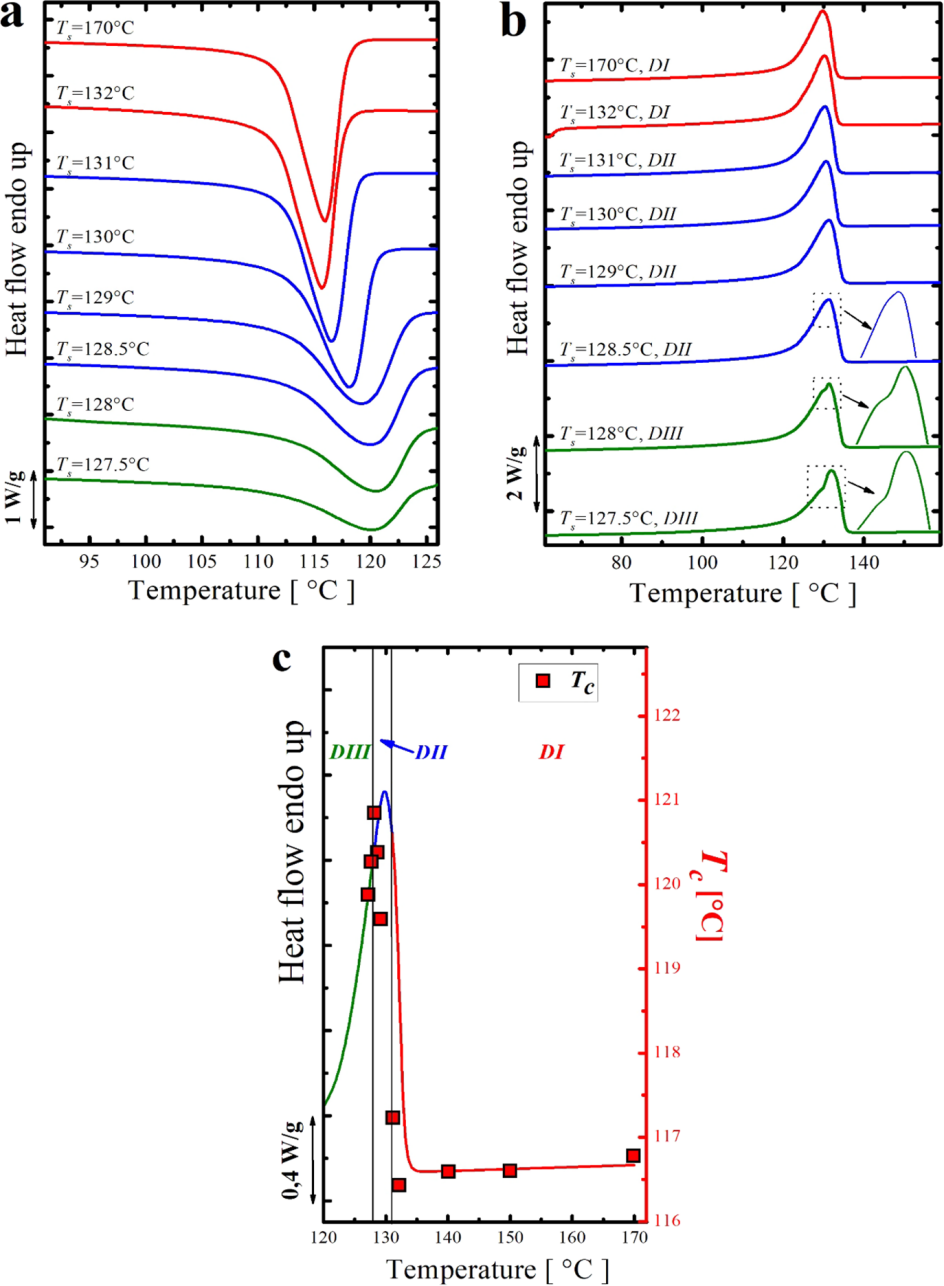
(a) DSC cooling scans (at 10 °C/min) of the neat HDPE-1 after 5 min at the indicated *T*_s_, (b) heating scans (at 10 °C/min) after the cooling runs shown in (a), and (c) representation of the SN *domains* for the neat HDPE-1 superimposed on the standard DSC heating curve. Red squares represent the *T*_c_ (right-hand-side *y*-axis) as a function of *T*_s_ (*x*-axis).

Figure 3a,b shows DSC cooling and heating scans obtained after self-nucleating the neat HDPE-1 at different SN temperatures (*T*_s_). In Figure 3c, the standard DSC heating curve of the neat HDPE-1 is plotted together with the crystallization temperatures (*T*_c_) recorded after different SN treatments, and the borders between the three characteristic SN *domains* are indicated as vertical lines. For clarity, DSC curves from different SN *domains* are plotted in different colors (red for *Domain I*, blue for *Domain II*, and green for *Domain III*) as suggested by Müller et al.^17,18^

The neat HDPE-1 presents a classical SN behavior with three SN *domains.* By applying *T*_s_ in the range of 132–170 °C, both crystallization and melting traces are unchanged, and the *T*_c_ recorded was 116 °C. This temperature range (i.e., 132 °C and above) corresponds to *Domain I* (or *DI*) in which only high-temperature-resistant heterogeneities/impurities are responsible for the obtained crystallization behavior. By lowering *T*_s_ in the range of 131–128.5 °C, a gradual increase in the *T*_c_ values upon decreasing *T*_s_ values is obtained, a behavior that corresponds to *Domain II* or the self-nucleation *Domain* (i.e., *DII*). In parallel to that, no changes in the melting characteristics have been recorded while the sample is in *DII* (see Figure 3b). The maximum increase in the crystallization temperature *T*_c_ without inducing any change in the melting behavior was 128.5 °C; that is the ideal self-nucleation temperature (*T*_s ideal_).

With the help of Figure 3c (the superposition of the *T*_c_ vs *T*_s_ value on top of the DSC melting scan of the neat HDPE-1), we can (i) say that the *melt memory domain* (*DIIa*) does not exist and (ii) deduce that the *DII* in the present case is only a *self-seeding domain* (*DIIb*), in which the observed SN nucleation behavior and the increase in *T*_c_ are only due to some crystal fragments existing in the polymer melt.^18,40^ The width of this obtained *self-nucleation domain* is 2.5 °C. As we mentioned previously, to the best of our knowledge, no clear agreement on the presence/absence as well as the width of the *DII* of HDPE has been achieved. For instance, in the work of Trujillo et al.,^20^ a total absence of *DII* was observed, while Carmeli et al.^7^ showed a *self-nucleation domain* of 1.5 °C width in their HDPE. This is probably because they are different HDPE samples, and each sample has a characteristic number of heterogeneities that depends on the catalytic debris content and other types of impurities present that can act as heterogeneous nuclei.

A further decrease in the applied *T*_s_ below 128.5 °C leads to the *self-nucleation and annealing domain* (*DIII*), where clear changes in the melting behavior of the HDPE-1 (step (5) of the thermal protocol described in the experimental section) are observed in parallel with the gradual increase in the *T*_c_. At *T*_s_ below 128.5 °C, the sample undergoes partial melting; hence, the remaining unmolten crystals will thicken (during the annealing process for 5 min at *T*_s_), resulting in an additional melting peak at a higher temperature.

#### 3.3.2 Self-Nucleation of the HDPE-1 in the 90/10 PS/HDPE-1 Blend

**Figure 4:**
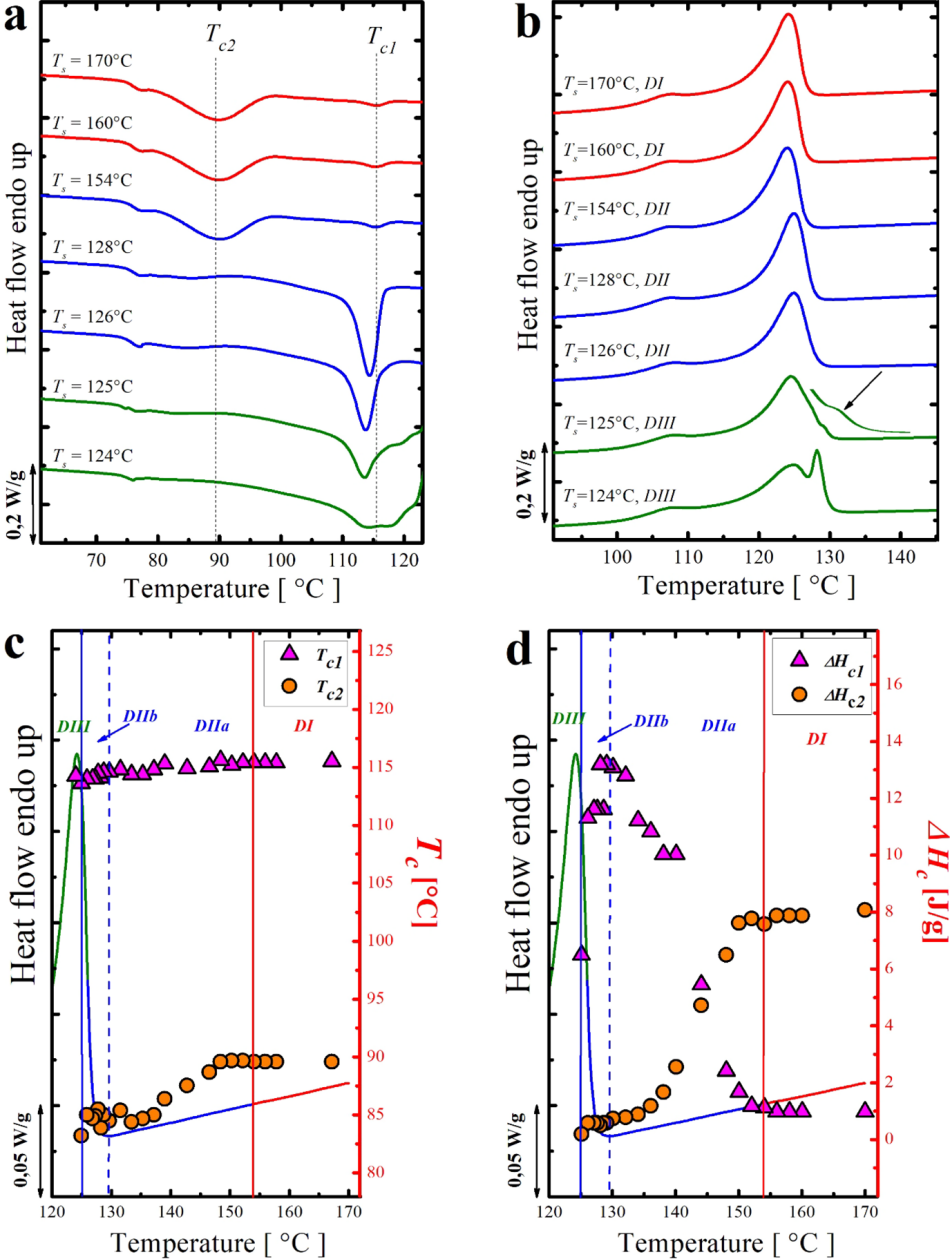
(a) DSC cooling scans (at 10 °C/min) of the 90/10 PS/HDPE-1 blend after 5 min at the indicated *T*_s_, (b) heating scans (at 10 °C/min) after the cooling runs shown in (a), and (c,d) collection of *T*_c_(s) and Δ*H*_c_(s) as a function of the employed *T*_s_ (*x*-axis) superimposed on top of the standard DSC melting trace.

Figure 4a shows the DSC cooling scans after self-nucleation of the HDPE-1 minor phase within the 90/10 PS/HDPE-1 at the indicated *T*_s_, while Figure 4b shows the subsequent heating scans. As we described previously, when the 90/10 PS/HDPE-1 blend is cooled from the isotropic melt, the HDPE-1 phase undergoes fractionated crystallization where two major peaks *T*_c1_ and *T*_c2_ are observed, at 115.6 and 90 °C, respectively. To avoid such fractionated crystallization behavior, several strategies have been applied, such as the addition of nucleating agents (NA) and the application of self-nucleation treatment.^18,23,31,41^

In the present 90/10 PS/HDPE-1 blend and at *T*_s_ higher than 154 °C, no appreciable changes in the crystallization features (*T*_c_(s), enthalpies, shape of the exotherms, and proportion or relative magnitude of each crystallization peak) are observed. This temperature range (170–155 °C) corresponds to the *complete melting domain* (*DI*).

Surprisingly, starting from a very high *T*_s_ (namely, 154 °C) down to 126 °C (*DII*), clear changes in the crystallization behavior are recorded, and the enthalpy of the high crystallization peak *T*_c1_ starts to increase at the expense of the low crystallization peak *T*_c2_ (see Figure 4a,d). In this range of temperatures, the thermal treatment applied during SN created/injected some self-nuclei (in the temperature range of 154–129 °C, which is the *melt memory domain* (*DIIa*)) and self-seeds (from 129 to 126 °C, corresponding to the *self-seeding domain* (*DIIb*)) inside the HDPE-1 droplets. The subdivision of the *DII* and the edge between *DIIa* and *DIIb*, which are shown in Figure 4c,d, are defined on the basis of the observed changes in the crystallization and melting behavior, as well as on the DSC melting endotherm of the HDPE-1 (after cooling from the standard melt). For instance, the upper limit of *DIIa* is defined as the *T*_s_ value at which changes in the crystallization behavior are observed, while the lower limit is fixed at the temperature at which all crystals are molten (the point where the DSC melting trace reaches the baseline). On the other hand, in the temperature range of *DII* (154–126 °C), no noticeable changes have been observed in the melting behavior. For the first time, HDPE-1 shows a *self-nucleation domain* (*DII*) with a width of 28 °C and an upper limit well above its equilibrium melting point *T*_m_° (30 °C above the *T*_m_ of the dispersed HDPE-1 droplets).

The lowest *T*_s_ temperature at which the maximum change in the crystallization behavior (in the magnitude of the two main crystallization peaks *T*_c1_ and *T*_c2_) is recorded was 126 °C. This is the ideal self-nucleation temperature (*T*_s ideal_), and at this SN temperature, most of the HDPE-1 droplets crystallized at a temperature identical to the one of bulk HDPE-1, and only a very small portion of the droplets crystallized at a higher supercooling (i.e., at 75 °C). This small droplet population that crystallizes displaying very small magnitude exotherms at peak crystallization temperatures close to 75 °C needs even lower *T*_s_ values to become self-nucleated. This could be related to the droplets’ small dimensions. We have not studied in detail their behavior, as they represent a minor fraction of the total crystallization enthalpy of the HDPE-1 phase.

Below *T*_s_ = 126 °C, more droplets undergo self-nucleation, and the area of the exotherm located at *T*_c2_ continues to decrease gradually until it disappears completely at *T*_s_ = 124 °C. In parallel, clear changes in the final heating scans have occurred where the appearance of another small melting peak at a higher temperature is observed after SN at *T*_s_ of 125 and 124 °C. The additional small endotherms observed after SN at 125–124 °C (indicated by an arrow in Figure 4b) are due to the melting of the crystals that were annealed and thickened during 5 min at *T*_s_. From the previously mentioned observation, it can be concluded that the *self-nucleation and annealing domain* (*DIII*) for most of the HDPE-1 droplets is located at *T*_s_ values below 126 °C.

To confirm that the peculiar SN behavior was not related to any change occurring in the HDPE-1 during melt blending, the HDPE-1 phase in the 90/10 PS/HDPE-1 blend was recovered by extracting PS with hot toluene and separating the HDPE-1 phase via centrifugation. The recovered material was then subjected to SN study. The obtained results are shown in Figure S5. It is clear that the recovered HDPE-1 exhibits SN behavior similar to the one of the neat HDPE-1 with a very narrow *DII* (only 0.5 °C width), which starts at a low *T*_s_ (129 °C). These results suggest that the peculiar SN behavior observed in Figure 4 is due to some changes in the state of the interface between PS and HDPE-1 in the blend. Further explanation will be given in the Discussion section.

Results of the SN of the HDPE-1 dispersed phase in the 85/15, 80/20, 70/30, and 50/50 PS/HDPE-1 blends (blends with higher HDPE-1 contents) are shown in Figures S6–S9, respectively. On the other hand, Figures S10–S15 present the SN of the HDPE droplets in other systems (other immiscible blends prepared using different HDPE grades and/or different amorphous matrices), which were tested to assess the generality of this observation. All the tested blends exhibited similar SN behavior to the 90/10 PS/HDPE-1, in particular with *DII* starting at a very high *T*_s_ (always ≥150 °C).

After examining the self-nucleation of HDPE droplets in immiscible blends with amorphous matrices, a semicrystalline matrix is used, and the SN behavior of the dispersed HDPE-2 minor phase is investigated.

#### 3.3.3 Self-Nucleation of the HDPE-2 in the 90/10 Nylon 6/HDPE-2 Blend

**Figure 5:**
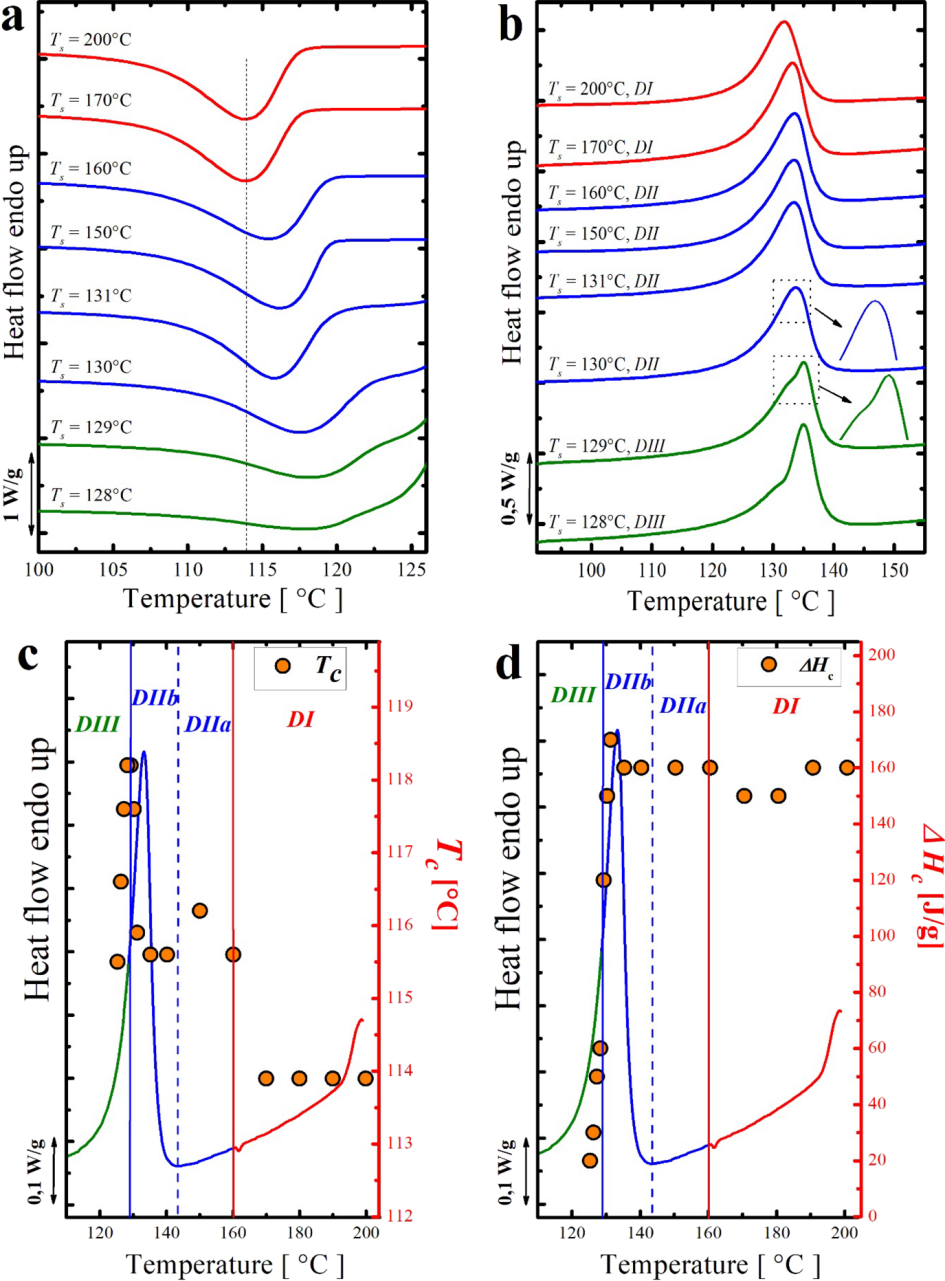
(a) DSC cooling scans (at 20 °C/min) of the 90/10 Nylon 6/HDPE-2 blend after 5 min at the indicated *T*_s_, (b) heating scans (at 20 °C/min) after the cooling runs shown in (a), and (c,d) collection of *T*_c_ and Δ*H*_c_ as a function of the employed *T*_s_ (*x*-axis) superimposed on top of the standard DSC melting trace.

Figure 5a,b presents DSC cooling and heating scans of a 90/10 Nylon 6/HDPE-2 blend at the indicated *T*_s_ values. Meanwhile, the collection of the *T*_c_ and Δ*H*_c_ as a function of the applied *T*_s_ is superimposed on top of the standard DSC melting endotherm of the HDPE-2 phase and shown respectively in Figure 5c,d.

At *T*_s_ > 160 °C, both crystallization and melting of the HDPE-2 minor phase are invariant. In this temperature range, which represents *DI*, the crystallization behavior is controlled only by high-temperature-resistant heterogeneities.

Again, the SN *DII* began at a very high *T*_s_ value for a linear polyethylene sample, namely, 160 °C. In fact, by lowering *T*_s_ to 160 °C, the *T*_c_ of the HDPE-2 phase increases from 114 to around 115.5 °C. By reaching the lower limit of the *DII* (at 130 °C), a *T*_c_ of about 117.5 °C was achieved. Meanwhile, no noticeable change in the melting behavior is observed.

*DIII* (*self-nucleation and annealing domain*) starts at *T*_s_ values lower than 130 °C. In this temperature range and even though the *T*_c_ is still increasing with the decrease in *T*_s_, the sample is only partially molten, and the unmolten crystal fragments experience annealing, become thicker, and result in a second melting peak at higher temperatures in the final heating scan.

## 4 Discussion

In this part, the peculiar self-nucleation behavior of the HDPE droplets and the injection of self-nuclei at temperatures well above the equilibrium melting point will be considered.

As we mentioned previously, after self-nucleating the recovered HDPE-1 (from 90/10 PS/HDPE-1), it was found that its behavior is similar to the one of neat HDPE-1. This means that the observed SN behavior in the blends could be attributed to the existence of a nucleating interface with the matrix and, most probably, to the roughness of the surface.

In a previous work, Müller et al.^42^ investigated the crystallization of the 80/20 PLA/PCL immiscible blend from the glassy state. They analyzed the cold crystallization of PLA after crystallizing the PCL phase at different *T*_c_ values. They reported a direct correlation between the crystallinity degree of the dispersed PCL droplets and the cold crystallization rate of PLA, in which the higher the crystallinity degree of PCL, the lower the PLA cold crystallization temperature *T*_cc_. A higher crystallinity degree of the PCL droplets means more shrunk droplets, which induced some additional roughness and stress at the PLA/PCL interface and led to a faster PLA nucleation.

In addition, Gałeski and Bartczak^43^ reported significant changes in the surface state in immiscible blends (or in a sandwich of two immiscible components) after crystallizing one component. The interface between the two components shifts from flat to an interface full of cavities and grooves. The reason behind the modification of the interface is the shrinkage of the crystallizing components (when it converts from a melt to the semicrystalline state), which induces significant deformation of the interface and pushes the melt of the second component to flow and fill those grooves and cavities. As a consequence, more surface area and more contact between the two components will be obtained. On the other hand, the appearance of a new rough surface will in principle favor surface nucleation and accelerate the nucleation process.^7,15^

It should be noted that rough or wrinkled surface topographies resulting from the so-called buckling instabilities are indeed obtained in a variety of systems, from electrospun polymer fibers^44^ to films on a substrate,^45^ as a consequence of a deformation mismatch between two phases (related for instance to thermal expansion or shrinkage).

For the PS/HDPE-1 immiscible blends prepared here, during cooling from the melt (step (2) in the SN protocol), the HDPE-1 droplets undergo an initial crystallization process starting from a temperature of about 119 °C. The HDPE-1 droplet surface will then become rough, as a consequence of lamellae formation. However, this roughness cannot be imprinted on the PS surface because of the low temperature, close to its *T*_g_, and related high viscosity. On the other hand, upon heating from the standard state to *T*_s_ (step 3 of the SN protocol) the PS becomes less viscous and can adhere to the HDPE-1, replicating its rough surface topography.^44^ We assume that the obtained additional roughness can be erased only by heating the sample to higher temperatures where the HDPE-1 droplets are fully molten and the PS component is fluid enough.

As SN is based on the partial/complete melting during the subsequent heating scan (step (3) of the SN thermal protocol), the applied *T*_s_ will have a major importance because it will determine if HDPE-1 droplets are fully molten or not and hence if the PS/HDPE-1 interface is fully relaxed and smooth or the opposite. *T*_s_ will also affect the crystalline memory of the HDPE-1 droplets and the state of the polymer chains (i.e., the presence or absence of any type of order), especially inside the formed cavities and grooves at the interface.

**Figure 6:**
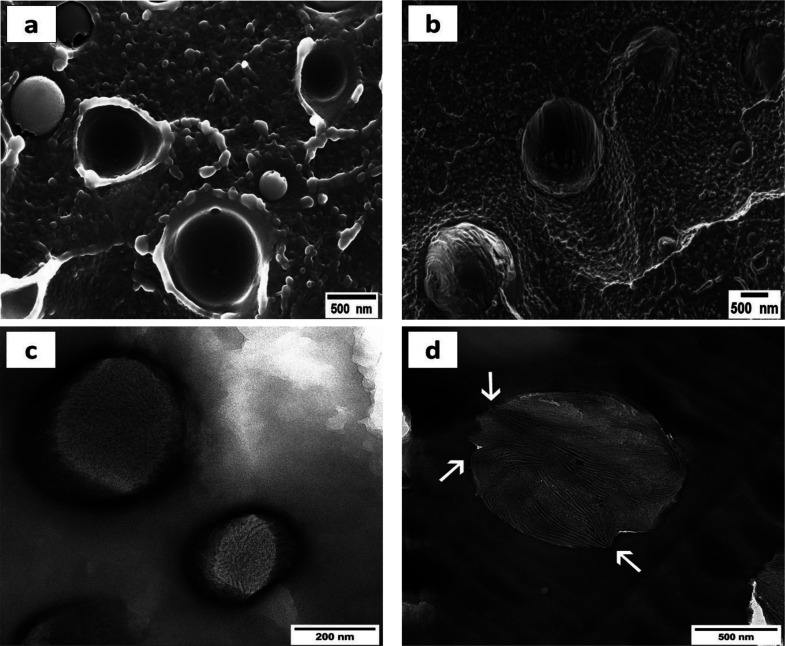
(a,b) SEM micrographs and (c,d) TEM micrographs of the SN 90/10 PS/HDPE-1 blend; (a,c) SN at 170 °C and (b,d) SN at 140 °C. The arrows show points from which some HDPE-1 crystalline lamellae start. The applied thermal protocol before this SEM/TEM analysis is shown in Figure S1.

Figure 6a,b shows SEM micrographs of the cryogenically fractured surface of the 90/10 PS/HDPE-1 sample after self-nucleation at *T*_s_ values of 170 and 140 °C, respectively. The SEM micrographs were taken at room temperature (after step (4) of the SN thermal protocol). As expected, a clear change in the interface state and the surface roughness of the two components is observed. At *T*_s_ = 170 °C (*DI*), the HDPE-1 droplets are fully molten, the viscosity of the PS is low enough, the surfaces of the PS and HDPE-1 are fully relaxed and thus the interface between PS and HDPE-1 is smooth (see Figure 6a). On the contrary, at *T*_s_ within *DII* (140 °C), the interface between PS and HDPE-1 remains deformed and rough; therefore, it helps in the nucleation of the HDPE-1 droplets during the subsequent cooling scan. As a conclusion, severe changes of the interface between the two components occurred and lead to a very rough interface (Figure 6b). A second SEM micrograph to confirm the above observation is taken at 132 °C, a temperature in *Domain II* and closer to *T*_s ideal_, and shown in the Supporting Information (Figure S16).

Figure 6c,d shows TEM micrographs of the 90/10 PS/HDPE-1 self-nucleated at 170 and 140 °C, respectively. Figure 6d shows that by decreasing *T*_s_ to 140 °C, a temperature within *DII*, surface nucleation tends to become predominant, with the arrows pointing some spots at the PS/HDPE interface from where crystalline HDPE lamellae initiate.

To summarize, the interface roughening occurs as a consequence of PS filling the surface asperities of solid HDPE-1 during the heating stage to *T*_s_ in the SN experiment. If the *T*_s_ temperature is low enough, this rough topography of the PS surface is retained, because the high polymer viscosity prevents interface relaxation. These additional nucleation sites created at low *T*_s_ temperature are present on the PS-HDPE-1 interface during the following cooling step, and induce surface nucleation, i.e., faster crystallization. Vice versa, if the selected SN temperature is too high (e.g., within *Domain I* or higher *T*_s_ values of *Domain II*), the PS viscosity drops and the interfacial roughness can be erased within the time given for the self-nucleation step at that temperature.

On the basis of the previous observations, two main factors can be used to explain the observed peculiar SN behavior: (i) the above-mentioned change in the interface state and the induced surface roughness, which favor surface nucleation, and (ii) stabilization of some ordered structures inside the cavities and grooves at the interface. These trapped self-nuclei will be more resistant and require higher *T*_s_ to be fully erased; thus, a very large *DII* (together with the appearance of *DIIa*) is obtained.

For the case of the 90/10 Nylon 6/HDPE-2 blend, the situation is somehow different since the Nylon 6 crystallizes first so that this process will control the shape and roughness of the interface. In this case, the enhanced SN behavior is mainly attributed to the stabilization of self-nuclei (or other ordered structures) at the interface inside the cavities formed by Nylon 6 lamellae.

It is also apparent that the SN behavior should be affected by the specific surface area of the interface in the blends. However, the way that the specific surface area of the wrinkled interfacial topography varies with the applied thermal history is not known and cannot be easily disclosed.

### 4.1 Transition between SN *Domains*

**Figure 7:**
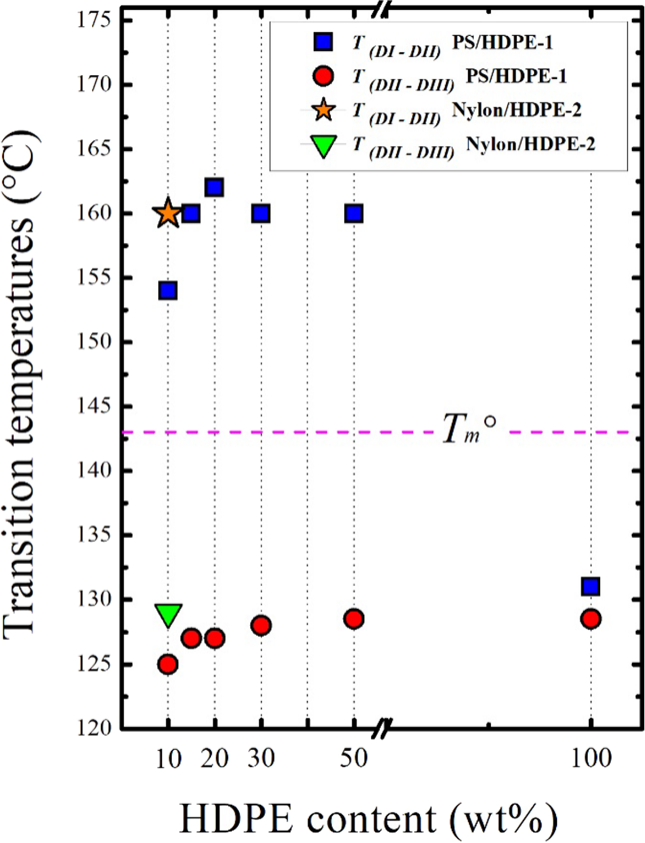
Transition temperatures of *DI–DII* and *DII–DIII* in the PS/HDPE-1 blends (with different HDPE-1 contents) as well as for the 90/10 Nylon 6/HDPE-2.

Figure 7 presents a collection of the *T*(*DI–DII*) (*T*_s_ at which the transition between *DI* and *DII* occurred) and *T*(*DII–DIII*) (*T*_s_ of the transition between *DII* and *DIII*) in all studied blends. All values are reported in Table 3.

**Table 3:** Recorded Transition Temperatures of *DI–DII* and *DII–DIII* in All Blends

blend	*T*_(_*DI–DII*) (°C)	*T*_(_*DII–DIII*) (°C)
neat HDPE-1	131	128
90/10 PS/HDPE-1	154	125
recovered HDPE-1	129	128
85/15 PS/HDPE-1	160	127
80/20 PS/HDPE-1	162	127
70/30 PS/HDPE-1	160	128
50/50 PS/HDPE-1	160	128.5
90/10 Nylon 6/HDPE-2	160	129
90/10 PS/HDPE-3	160	127
90/10 PMMA/HDPE-1	160	128
90/10 PETG/HDPE-1	160	126
90/10 PC/HDPE-1	152	126
90/10 PS-2/HDPE-1	152	124
90/10 PS-2/HDPE-4	152	124

It is clear that regardless of the content of HDPE in the blend as well as the type of the matrix (either amorphous or semicrystalline), the HDPE phase always exhibits a high melt memory temperature range, which is well above the equilibrium melting point. The obtained results indicate that the observed HDPE phase SN behavior is mainly attributed to the presence of foreign surfaces (i.e., PS or Nylon 6) and not to the size of the dispersed phase or to the produced morphology.

## 5 Conclusions

Dispersing HDPE into immiscible blends greatly enhances its self-nucleation capacity in comparison with neat HDPE. While neat HDPE only exhibits a very narrow *Domain II* characterized by only self-seeding (i.e., *DIIb*), HDPE droplets in PS or Nylon 6 matrices develop a very wide *Domain II* that includes both a *self-seeding domain* (*DIIb*) and a very wide *melt memory domain* (*DIIa*). The *Domain IIa* is so wide for the HDPE droplets in the blends that temperatures above *T*_m_° are needed to achieve the isotropic state or *Domain I*.

The much stronger melt memory exhibited by the HDPE phase in the blends occurs when HDPE is blended with several amorphous (like PS or PMMA) or even semicrystalline (Nylon 6) immiscible polymeric components. Therefore, we conclude that it is a general effect present in blends with dispersed HDPE phases. We demonstrated by extracting the HDPE in PS/HDPE blends that the material reverts back to the behavior of neat HDPE when it does not form part of a blend.

SEM and TEM experiments provided definite evidence that allows us to conclude that the enhanced self-nucleation effect in the HDPE droplets is due to an interfacial roughening effect. Such increased surface roughness creates protrusions and crevices (imaged by SEM) that can enhance self-nucleation and melt memory effects via a surface nucleation mechanism. The nucleation capacity of the interface was demonstrated by TEM images.

## Supporting Information Available

Thermal protocol used before SEM and TEM of the SN samples; SEM results of some other binary blends; SN nonisothermal (cooling and heating) analyses of other binary blends; table listing the recorded thermal transition of the HDPE in all the studied systems; SN at random *T*_s_ to check the morphological stability in the 90/10 PS/HDPE-1 blend; SN results of the recovered HDPE-1; SN of the HDPE-1 in the 85/15 PS/HDPE-1, 80/20 PS/HDPE-1, 70/30 PS/HDPE-1, and 50/50 PS/HDPE-1 blends; SN of the HDPE-3 in the 90/10 PS/HDPE-3; SN in 90/10 PMMA/HDPE-1; SN in 90/10 PS-2/HDPE-1; SN in 70/30 PS-2/HDPE-4; SN in 90/10 PETG/HDPE-1; SN in PC/HDPE-1; SEM micrograph of the 90/10 PS/HDPE-1 after SN at 132 °C (PDF)
