# Supplementary material for: Surface Roughness Enhances Self-Nucleation of High-Density Polyethylene Droplets Dispersed within Immiscible Blends
Source: Macromolecules. 2022 Feb 11;55(4):1412–23. doi: 10.1021/acs.macromol.1c02487 (PMC8874415; doi:10.1021/acs.macromol.1c02487)
Supplement: Supplementary file 1 — ma1c02487_si_001.pdf [file ma1c02487_si_001.pdf]

Surface roughness enhances self-nucleation of high-density polyethylene droplets dispersed within immiscible blends.

Seif Eddine Fenni [a], Maria Rosaria Caputo [b], Alejandro J. Müller [b,c,*], and Dario Cavallo [a,*]

[a] Dipartimento di Chimica e Chimica Industriale, Università degli studi di Genova, via Dodecaneso 31, 16146 Genova, Italy;

[b] Polymat and Department of Polymers and Advanced Materials: Physics, Chemistry and Technology, Faculty of Chemistry, University of the Basque Country UPV/EHU, Paseo Manuel de Lardizabal 3, 20018. Donostia-San Sebastián, Spain;

[c] IKERBASQUE, Basque Foundation for Science, Plaza Euskadi 5, 48009 Bilbao, Spain;

1. Self-nucleation preparation for the SEM analysis:

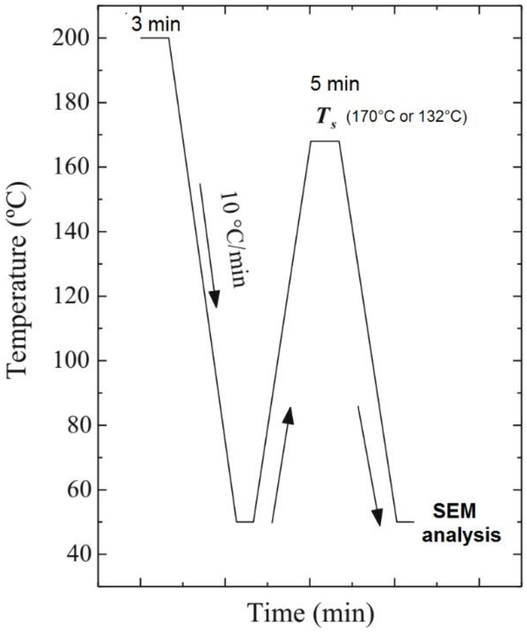

Figure S1. Thermal protocol applied to prepare samples for the SEM and TEM analysis shown in Figure 6.

2. SEM analysis:

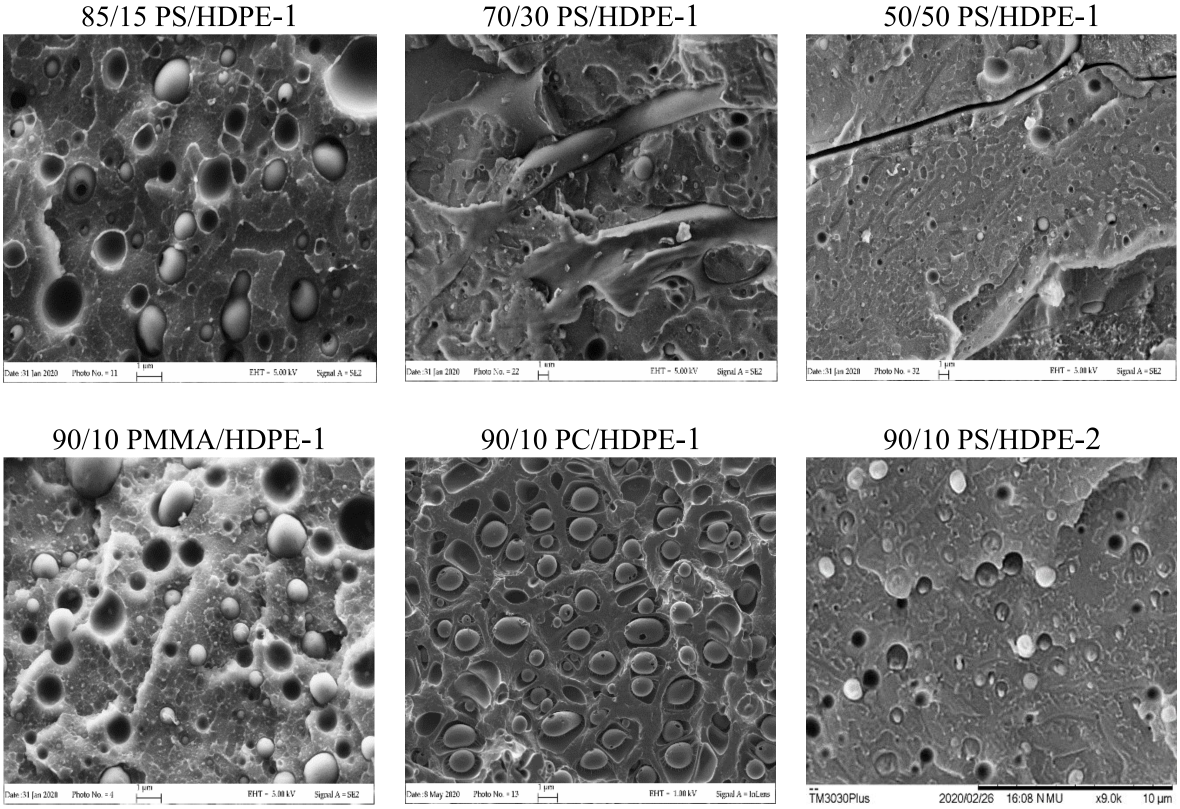

Figure S2. Morphologies of different binary blends.

3. DSC non-isothermal analyses:

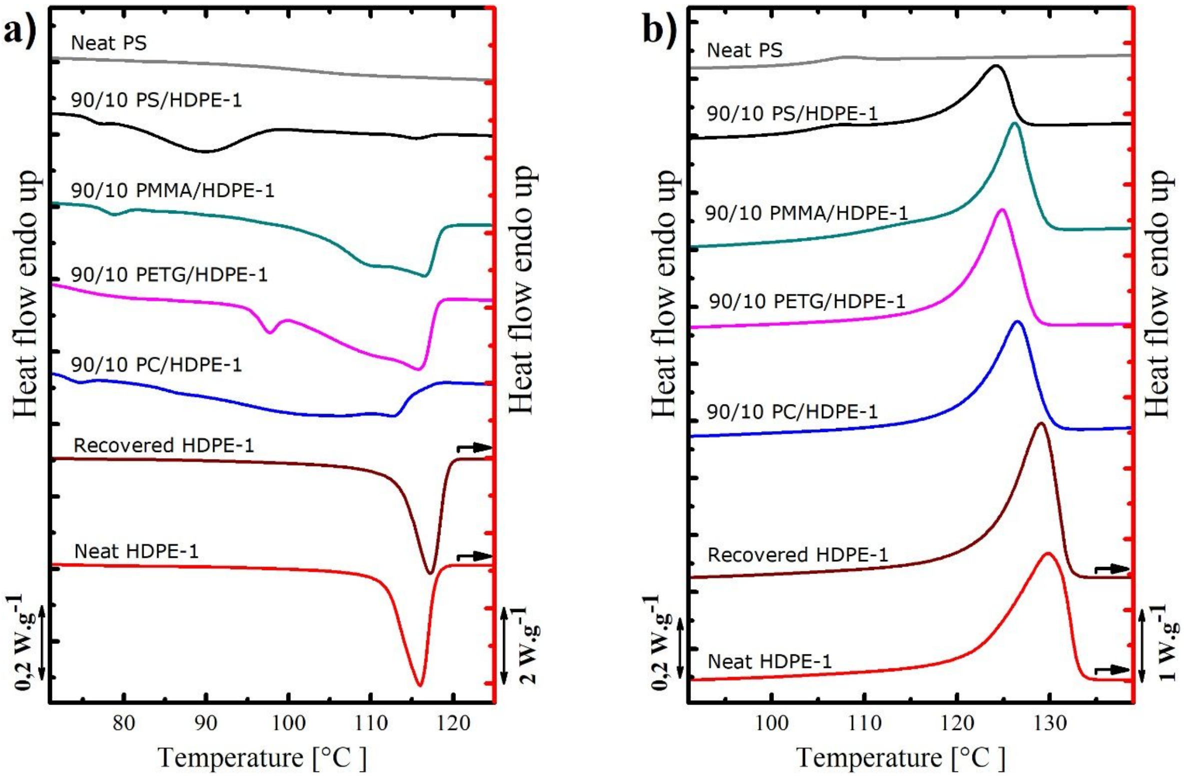

Figure S3. a) DSC cooling scans and b) subsequent DSC heating scans for the indicated blends at a cooling and heating rate of 10°C/min. The curves of neat HDPE-1 and 90/10 PS/HDPE-1 are added for the sake of comparison.

Table S1. Recorded T_c (s) and T_m at a scan rate of 10°C/min in all the investigated blends.

Blend	T_c (°C)		T_m (°C)
Neat HDPE-1	116		130
90/10 PS/HDPE-1	90	115.5	124
Recovered HDPE-1	117		129
85/15 PS/HDPE-1	94	117	126
80/20 PS/HDPE-1	98	117	126
70/30 PS/HDPE-1	117		128.5
50/50 PS/HDPE-1	116.5		128.6
90/10 Nylon 6/HDPE-2	116.5		131.5
90/10 PS/HDPE-3	82	118.5	127.5
90/10 PMMA/HDPE-1	110.5	116.5	126
90/10 PETG/HDPE-1	97.5	116	125
90/10 PC/HDPE-1	106	112.5	126.5
90/10 PS2/HDPE-1	90	114	123.5
90/10 PS2/HDPE-4	90	114.5	123.5

4. Check of the stability of the morphology during the SN analysis:

In order to check the In order to check the morphology stability and non-degradation of HDPE-1 in the 90/10 PS/HDPE-1, some repetitions of SN analysis at random T_s have been performed. Figure S4 confirms that no change in the crystallization behavior of the system which in turn confirm the stability (non-change) of the morphology of the immiscible blend and mainly the size of the HDPE-1 droplets (hence we can exclude the possibility of droplets coalescence and increase of the size). Figure S3 revealed as well that the HDPE-1 phase within the 90/10 PS/HDPE-1 blend does not receive any noticeable degradation.

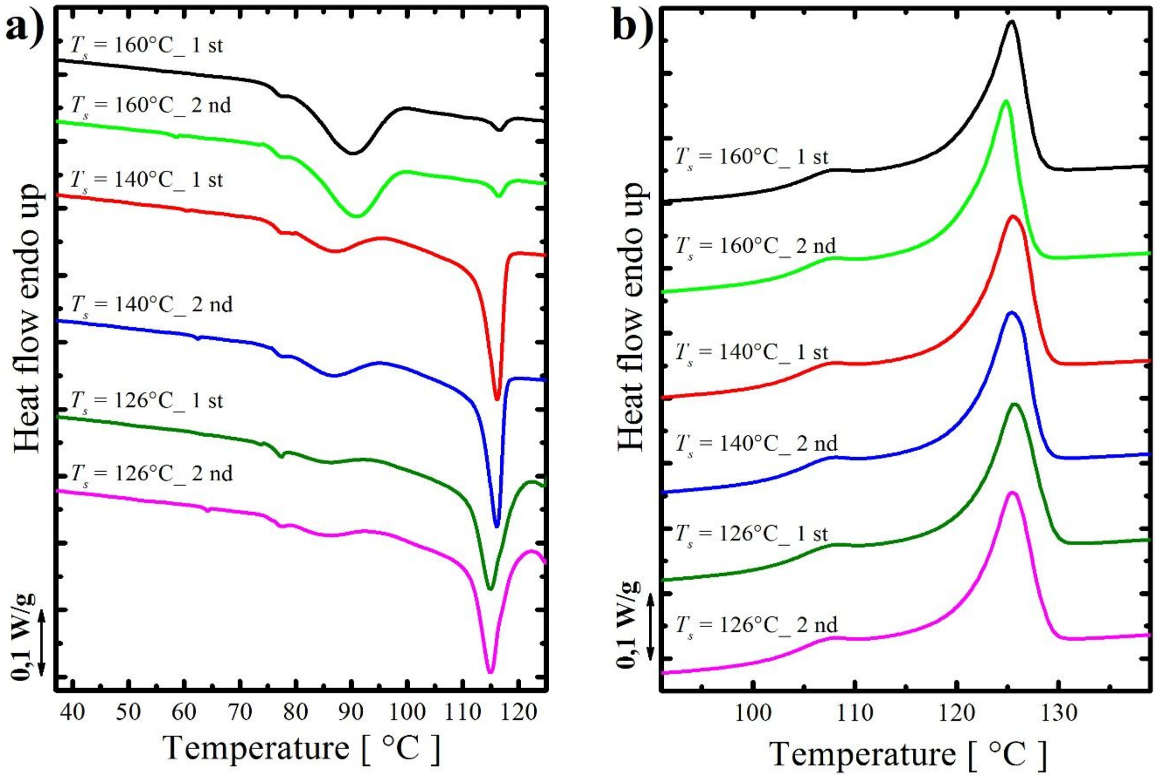

Figure S4. a) DSC cooling scans (at 10°C/min) of the 90/10 PS/HDPE-1 blend after 5 min at the indicated T_s; (b) Heating scans (at 10°C/min) after the cooling runs shown in (a).

5. Self-nucleation of the recovered HDPE-1:

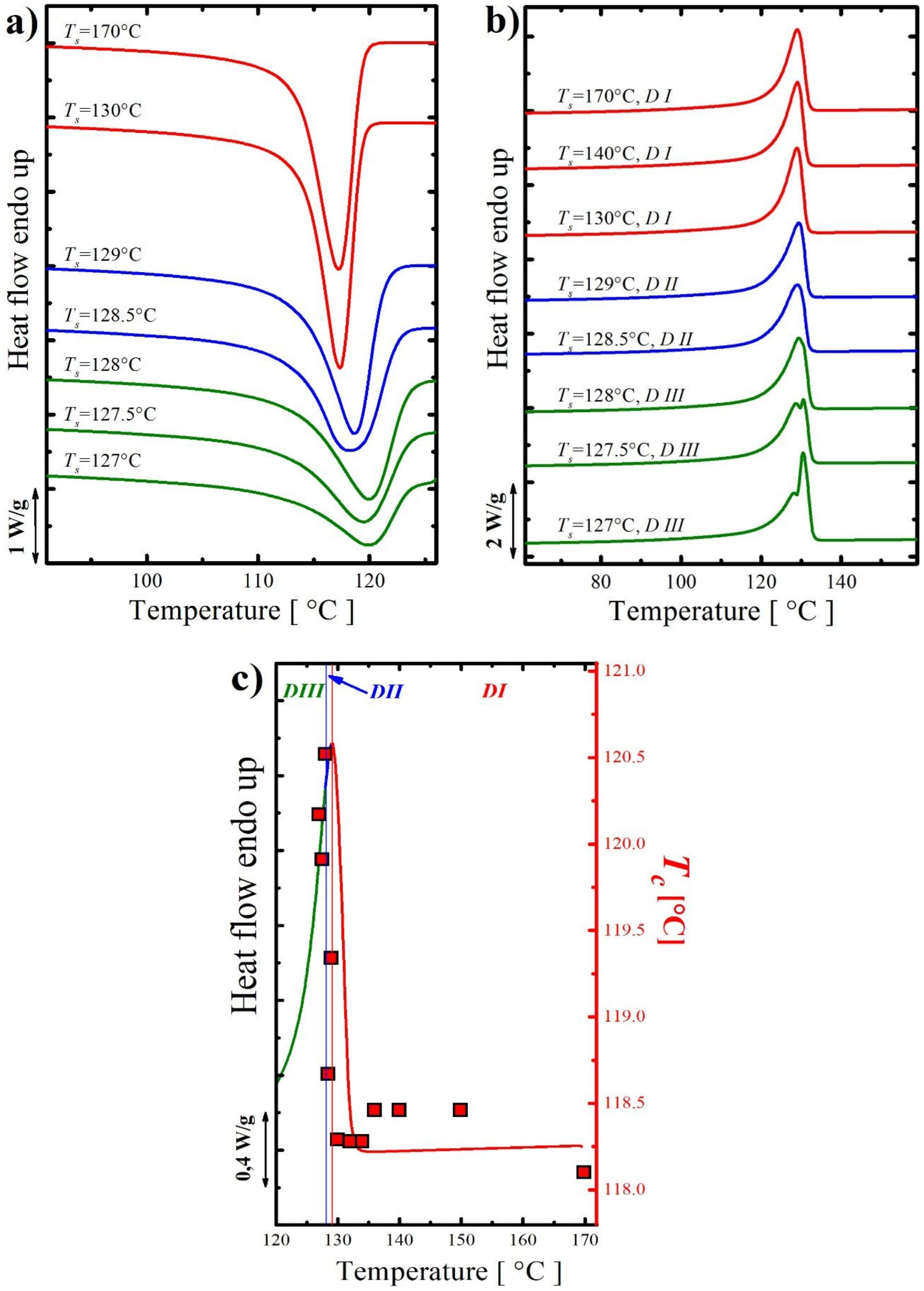

Figure S5. a) DSC cooling scans (at 10°C/min) of the recovered (recovered from the 90/10 PS/HDPE-1) HDPE-1 after 5 min at the indicated T_s; (b) Heating scans (at 10°C/min) after the cooling runs shown in (a); c) is a collection of T_c as a function of the employed T_s (x-axis) superimposed on top of the standard DSC melting trace.

6. Self-nucleation of the HDPE-1 in the 85/15 PS/HDPE-1 blend:

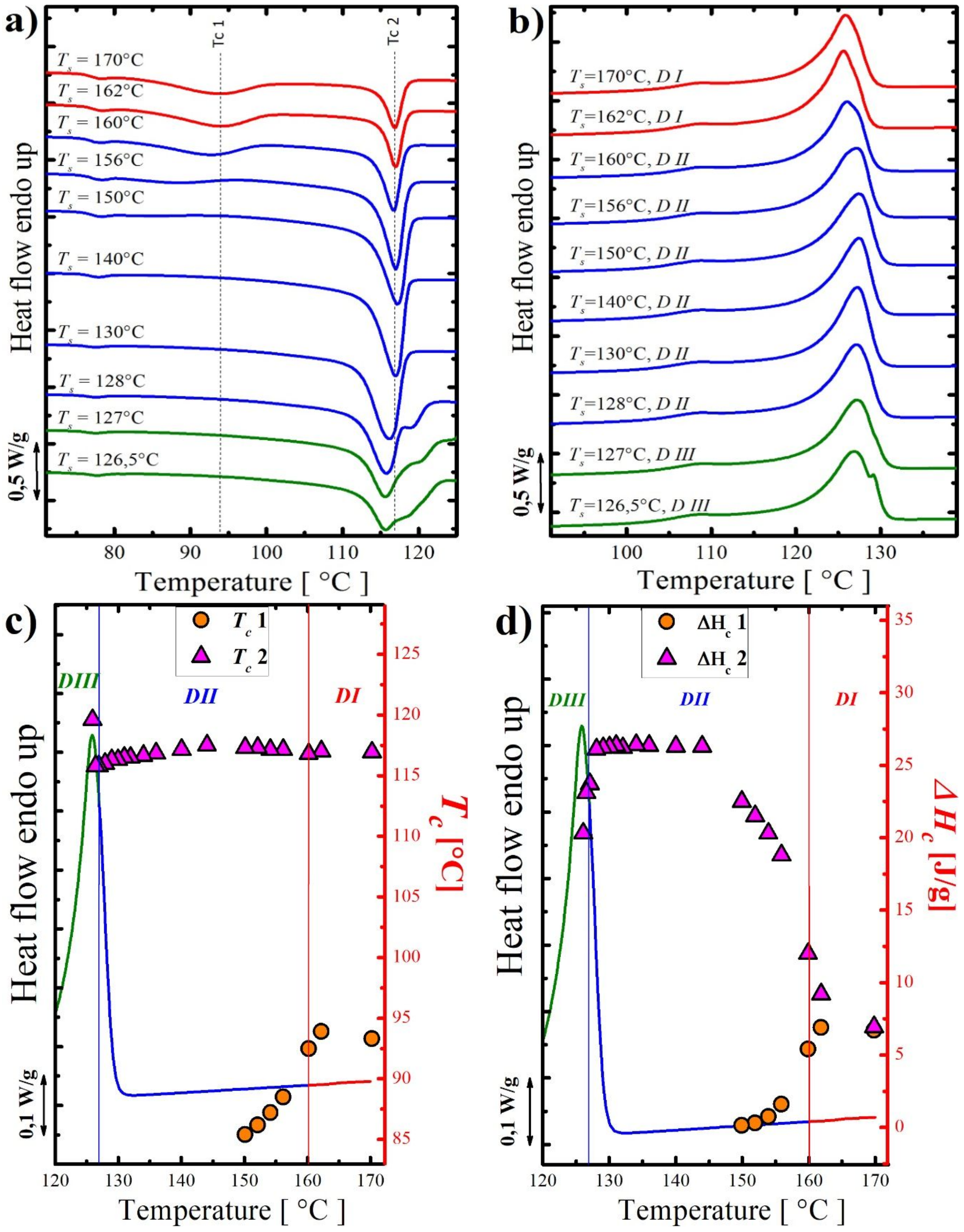

Figure S6. a) DSC cooling scans (at 10°C/min) of the 85/15 PS/HDPE-1 blend after 5 min at the indicated T_s; (b) Heating scans (at 10°C/min) after the cooling runs shown in (a); c), and d) are collection of T_c (s) and DH_c (s), respectively, as a function of the employed T_s (x-axis) superimposed on top of the standard DSC melting trace.

7. Self-nucleation of the HDPE-1 in the 80/20 PS/HDPE-1 blend:

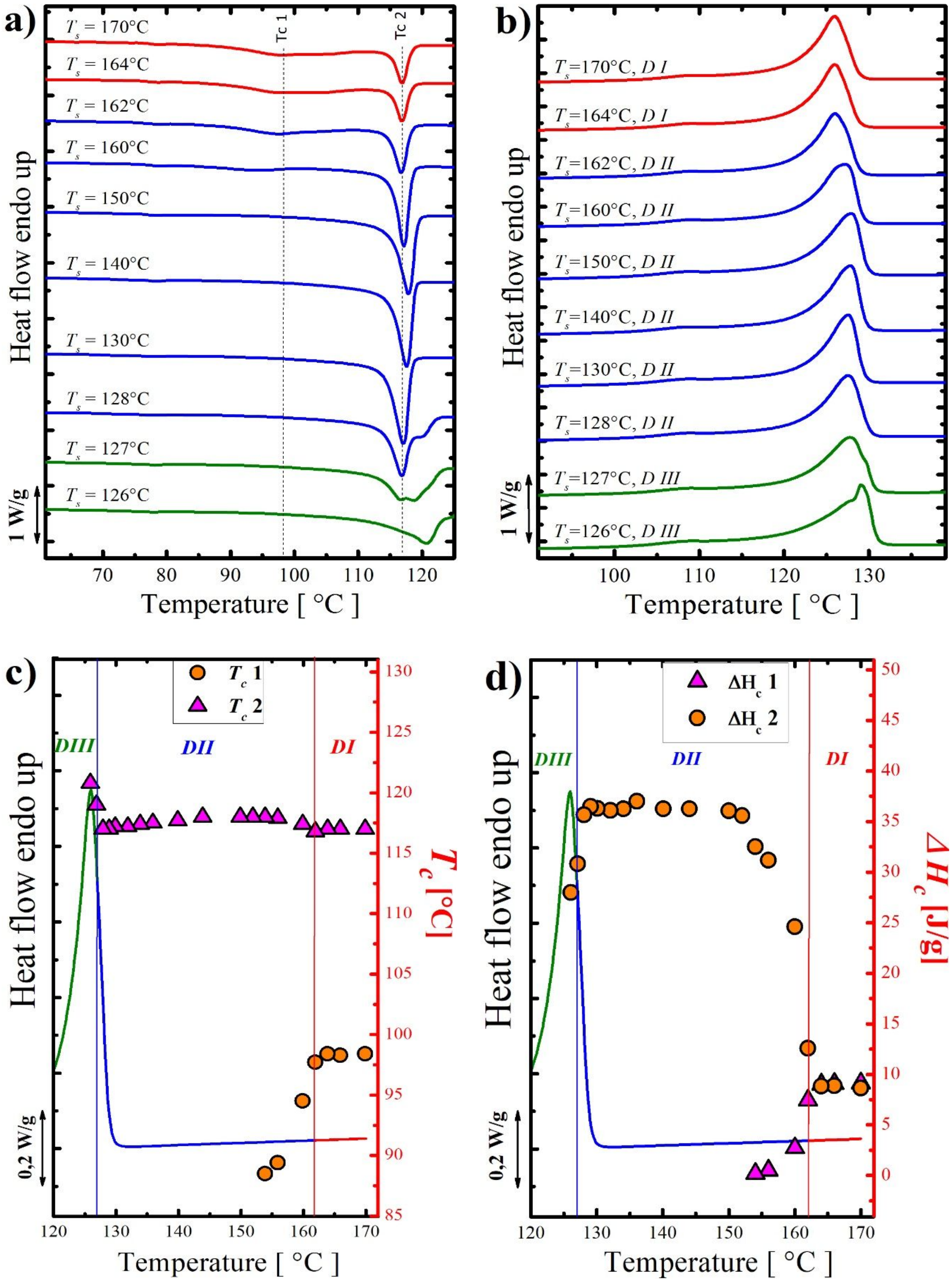

Figure S7. a) DSC cooling scans (at 10°C/min) of the 80/20 PS/HDPE-1 blend after 5 min at the indicated T_s; (b) Heating scans (at 10°C/min) after the cooling runs shown in (a); c), and d) are collection of T_c (s) and DH_c (s), respectively, as a function of the employed T_s (x-axis) superimposed on top of the standard DSC melting trace.

8. Self-nucleation of the HDPE-1 in the 70/30 PS/HDPE-1 blend:

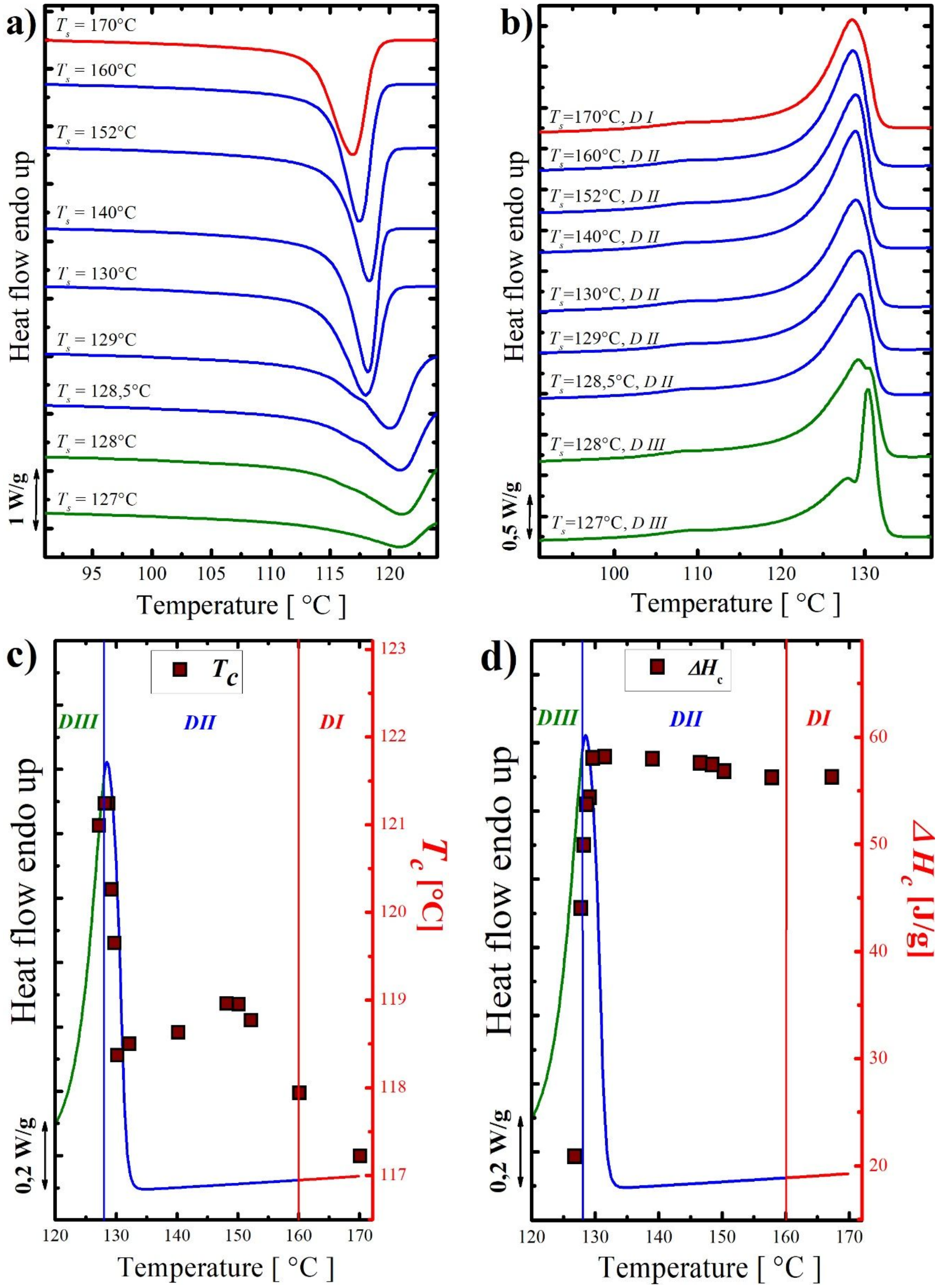

Figure S8. a) DSC cooling scans (at 10°C/min) of the 70/30 PS/HDPE-1 blend after 5 min at the indicated T_s; (b) Heating scans (at 10°C/min) after the cooling runs shown in (a); c), and d) are collection of T_c and DH_c , respectively, as a function of the employed T_s (x-axis) superimposed on top of the standard DSC melting trace.

9. Self-nucleation of the HDPE-1 in the 50/50 PS/HDPE-1 blend:

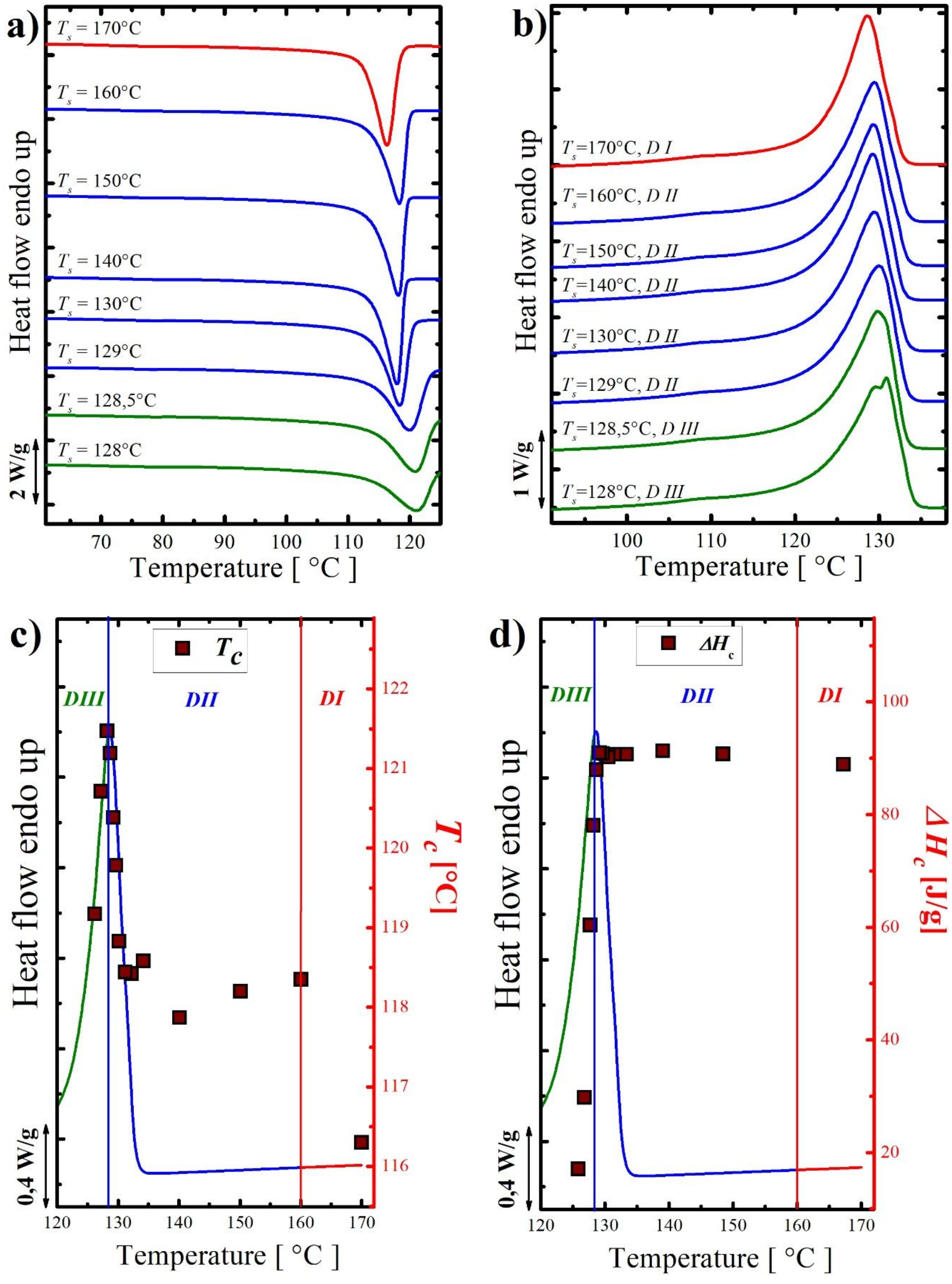

Figure S9. a) DSC cooling scans (at 10°C/min) of the 50/50 PS/HDPE-1 blend after 5 min at the indicated T_s; (b) Heating scans (at 10°C/min) after the cooling runs shown in (a); c), and d) are collection of T_c and DH_c , respectively, as a function of the employed T_s (x-axis) superimposed on top of the standard DSC melting trace.

10. SN using other types of HDPE or/and matrices

10.1. Self-nucleation of the HDPE-3 in the 90/10 PS/HDPE-3 blend:

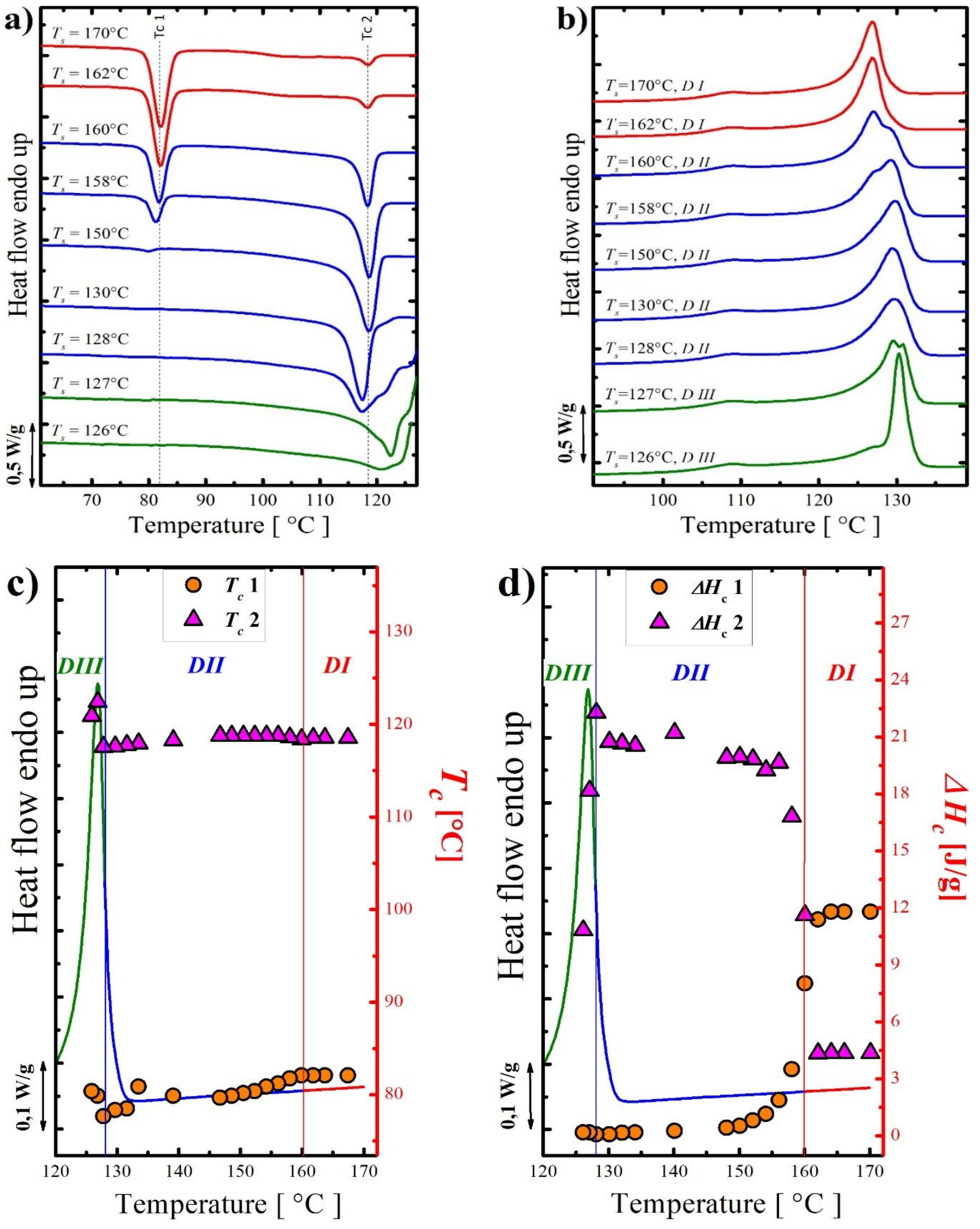

Figure S10. a) DSC cooling scans (at 10°C/min) of the 90/10 PS/HDPE-3 blend after 5 min at the indicated T_s; (b) Heating scans (at 10°C/min) after the cooling runs shown in (a); c), and d) are collection of T_c (s) and DH_c (s), respectively, as a function of the employed T_s (x-axis) superimposed on top of the standard DSC melting trace.

10.2. Self-nucleation of the HDPE-1 in the 90/10 PMMA/HDPE-1 blend:

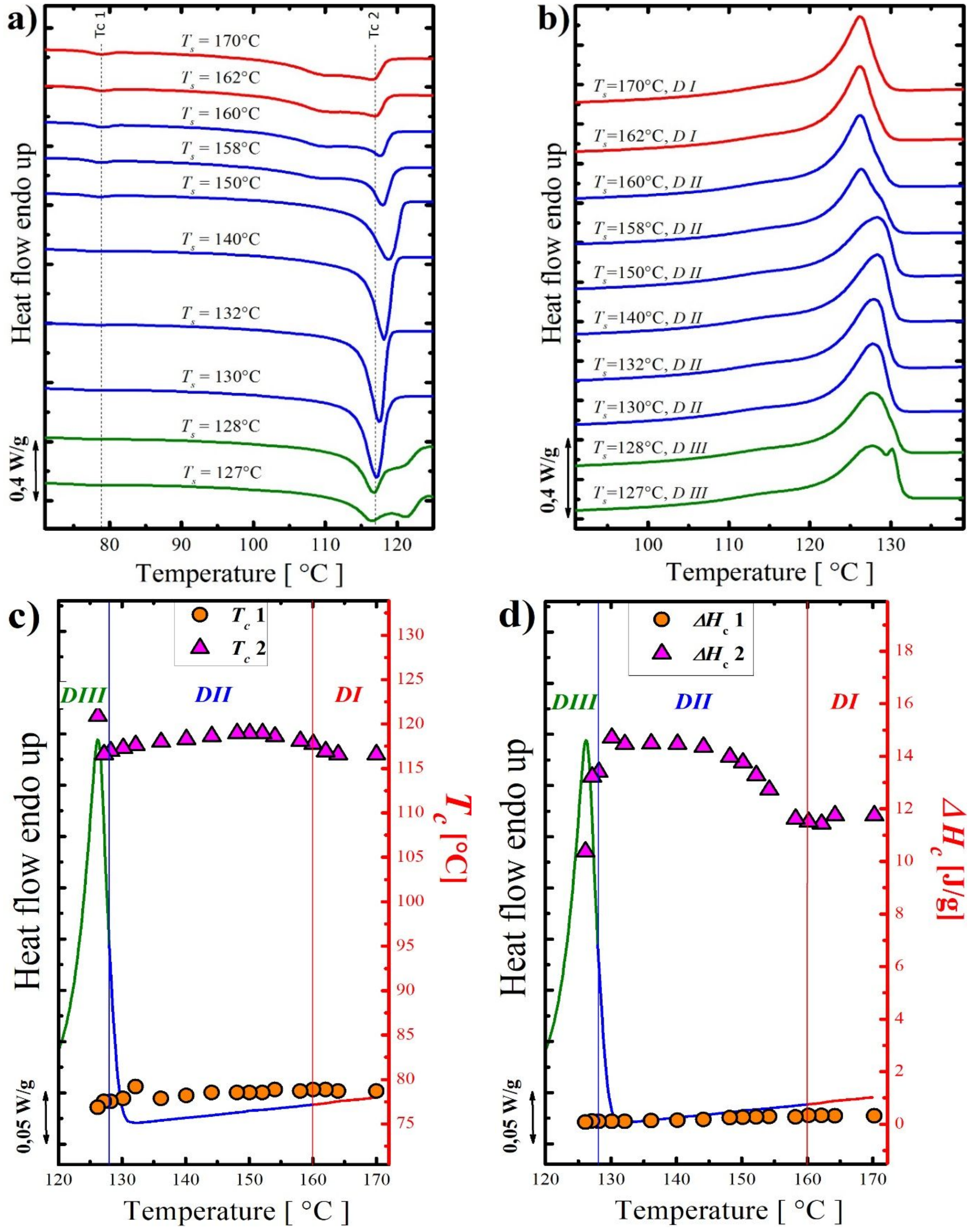

Figure S11. a) DSC cooling scans (at 10°C/min) of the 90/10 PMMA/HDPE-1 blend after 5 min at the indicated T_s; (b) Heating scans (at 10°C/min) after the cooling runs shown in (a); c), and d) are collection of T_c (s) and DH_c (s), respectively, as a function of the employed T_s (x-axis) superimposed on top of the standard DSC melting trace.

10.3. Self-nucleation of the HDPE-1 in the 90/10 PS-2/HDPE-1 blend:

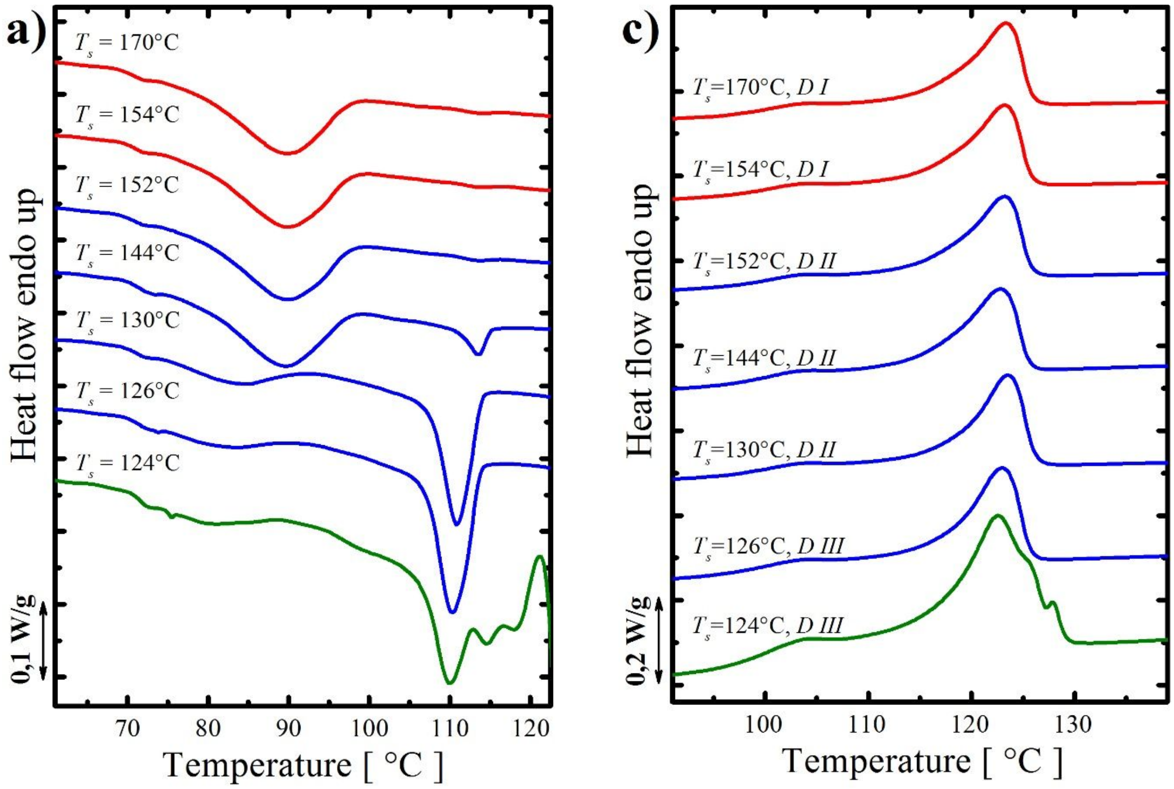

Figure S12. a) DSC cooling scans (at 10°C/min) of the 90/10 PS-2/HDPE-1 blend after 5 min at the indicated T_s; (b) Heating scans (at 10°C/min) after the cooling runs shown in (a).

10.4. Self-nucleation of the HDPE-4 in the 90/10 PS-2/HDPE-4 blend:

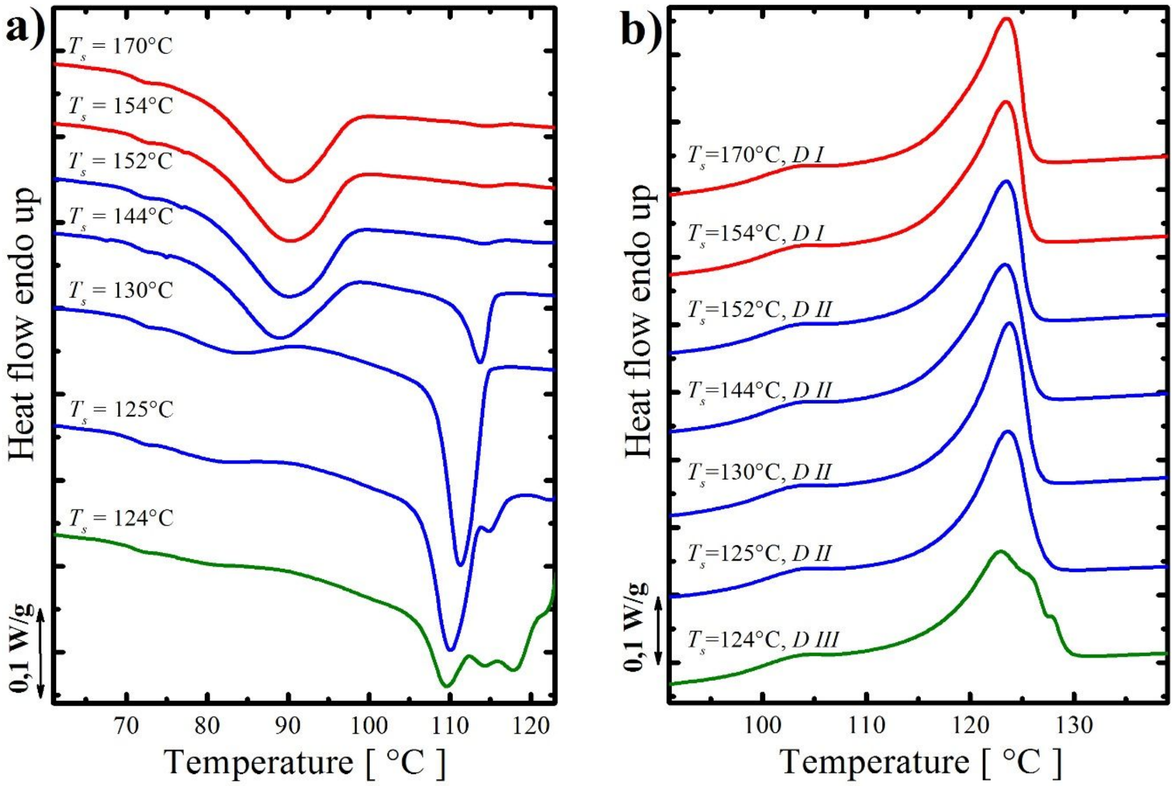

Figure S13. a) DSC cooling scans (at 10°C/min) of the 90/10 PS-2/HDPE-4 blend after 5 min at the indicated T_s; (b) Heating scans (at 10°C/min) after the cooling runs shown in (a).

10.5. Self-nucleation of the HDPE-1 in the 90/10 PETG/HDPE-1 blend:

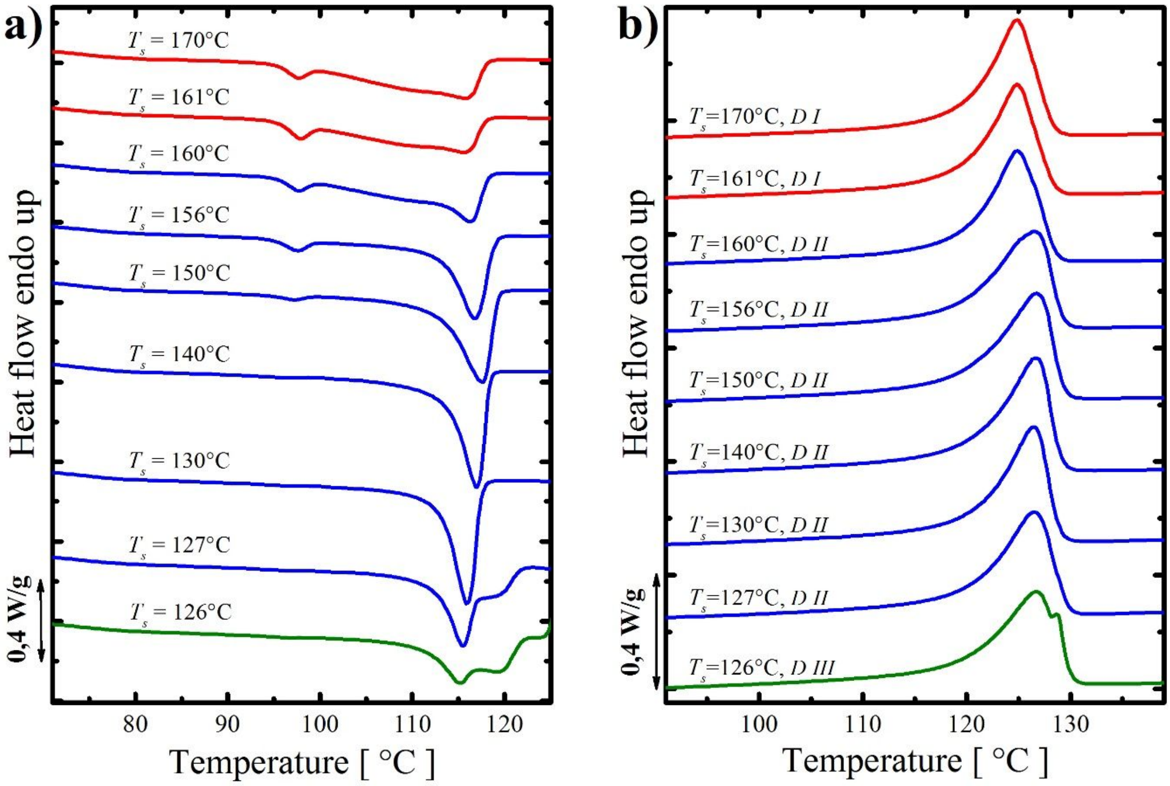

Figure S14. a) DSC cooling scans (at 10°C/min) of the 90/10 PETG/HDPE-1 blend after 5 min at the indicated T_s; (b) Heating scans (at 10°C/min) after the cooling runs shown in (a).

10.6. Self-nucleation of the HDPE-1 in the 90/10 PC/HDPE-1 blend:

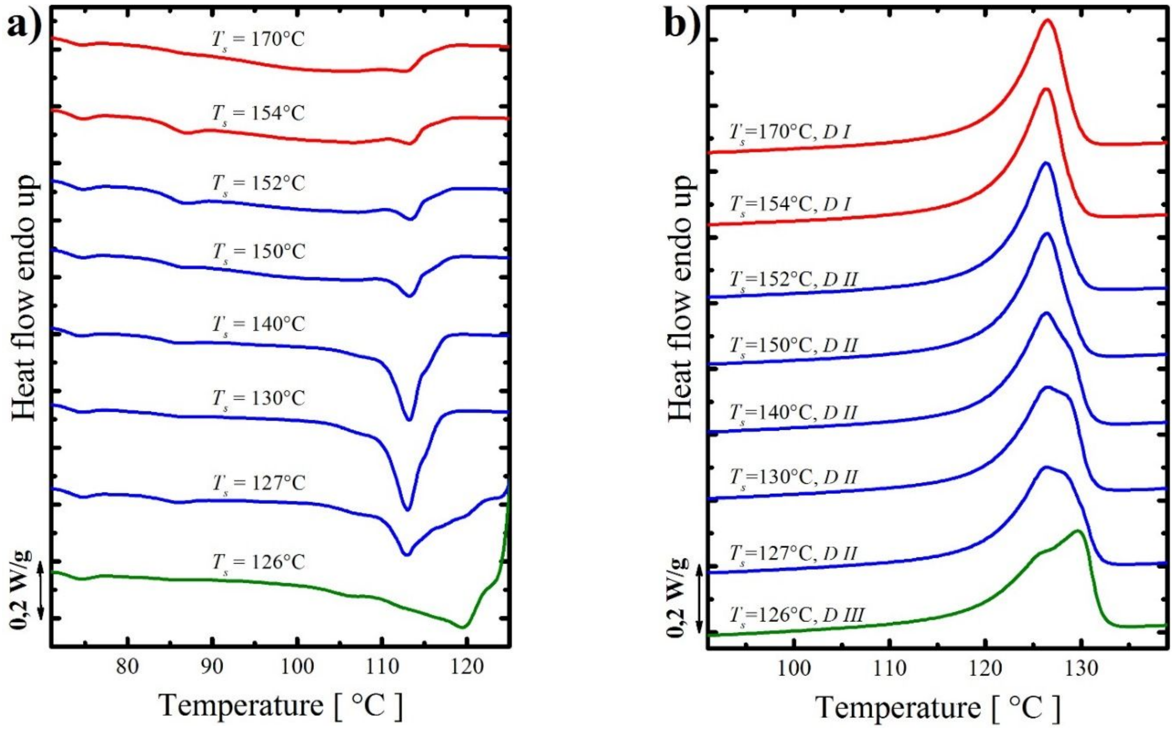

Figure S15. a) DSC cooling scans (at 10°C/min) of the 90/10 PC/HDPE-1 blend after 5 min at the indicated T_s; (b) Heating scans (at 10°C/min) after the cooling runs shown in (a).

11. SEM after SN at 132°C:

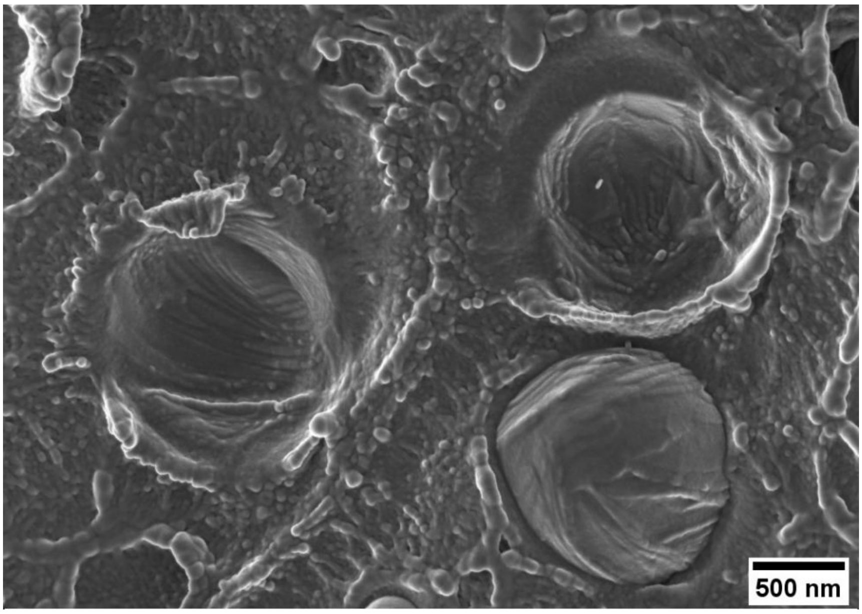

Figure S16. SEM micrograph of the 90/10 PS/HDPE-1 blend SN at 132°C.
